# Transcriptomic characterization of *Wolbachia* endosymbiont from *Leuronota fagarae* (Hemiptera: Psylloidae)

**DOI:** 10.20517/mrr.2024.84

**Published:** 2025-04-03

**Authors:** Douglas S. Stuehler Jr., Wayne B. Hunter, Jawwad A. Qureshi, Liliana M. Cano

**Affiliations:** ^1^Oak Ridge Institute for Science and Education ORISE, Department of Energy and the United States Department of Agriculture DOE/USDA, Agricultural Research Station ARS, Fort Pierce, FL 34945, USA.; ^2^United States Department of Agriculture USDA, Agricultural Research Station ARS, U.S. Horticultural Research Laboratory, Fort Pierce, FL 34945, USA.; ^3^Department of Entomology and Nematology, Southwest Florida Research and Education Center SWFREC, Institute of Food and Agricultural Sciences IFAS, University of Florida, Immokalee, FL 34142, USA.; ^4^Department of Plant Pathology, Indian River Research and Education Center IRREC, Institute of Food and Agricultural Sciences IFAS, University of Florida, Fort Pierce, FL 34945, USA.

**Keywords:** *Wolbachia*, *Leuronota fagarae*, wild lime psyllid, Citrus greening, Huanglongbing, microbiome, *Diaphorina citri*

## Abstract

**Aim:** *Wolbachia* species are among the most abundant intracellular endosymbionts of insects worldwide. The extensive distribution of Gram-negative *Wolbachia* among insects highlights their evolutionary success and close relationship with many insect host species. This study aimed to characterize a novel *Wolbachia* strain from the Wild Lime Psyllid, *Leuronota fagarae* (*L. fagarae*), to understand its evolutionary relationship with *Wolbachia* from psyllid pests like *Diaphorina citri*, the vector of Huanglongbing (HLB).

**Methods:** Wild-caught *L. fagarae* colonies from Florida, USA, were maintained on Zanthoxylum fagara. RNA was extracted from the salivary glands, heads, and whole bodies of male and female adult *L. fagarae*. Four cDNA libraries were sequenced using short read technology and de novo transcriptome assembly was performed. Multilocus sequence typing (MLST) of nine conserved loci and *wsp* gene analysis classified the strain’s phylogeny, while sequence mapping and functional annotation provided insight into host-microbe interactions.

**Results:** The new *Wolbachia* strain, designated *Wolbachia* endosymbiont of *Leuronota fagarae* (wLfag-FL), was assigned to supergroup B, showing relation to *Wolbachia* strains of other related psyllids. Transcriptome analysis identified 1,359 *Wolbachia* transcripts with 465 assigned functions encompassing metabolic and secretion system pathways. Ankyrin domain proteins and a partial bacterioferritin sequence were detected, suggesting nutritional provisioning roles.

**Conclusion:** The characterization of wLfag-FL expands the known *Wolbachia* host range and informs HLB-related pest biology. Its phylogenetic placement and transcript annotations offer insights into symbiotic interactions, potentially guiding environmentally safe pest control strategies targeting psyllid fitness and pathogen transmission.

## INTRODUCTION

The Wild Lime Psyllid, *Leuronota fagarae* (*L. fagarae*) Burckhardt (Hemiptera: Psylloidea), is an invasive species of Florida, USA, with origins traced to Paraguay, South America^[1]^. The first report of *L. fagarae* in the United States occurred in southern Florida when adults were discovered feeding and reproducing on citrus relative *Zanthoxylum fagara* (L.) Sarg. (Sapindales: Rutaceae)^[1]^. The 2001 discovery of *L. fagarae* in southern Florida led to the first classification of the species since its original description in 1988 from two locations in Paraguay^[2]^. Rolled leaf edges that provide safeguards for developing nymphs indicate a typical phenotype of *L. fagarae* feeding on citrus^[1]^. In Florida, the habitat range of *L. fagarae* overlaps with the Asan Citrus Psyllid (ACP, *Diaphorina citri* Kuwayama) in the transition zone from non-cultivated areas to areas of cultivated citrus trees. This proximity, as well as taxonomic relationship and potentially shared food source, provides an opportunity for the transfer of intracellular microbes through plant feeding^[3]^, such as *Wolbachia spp.*^[4]^.

The parasitism-mutualism balance linking host and endosymbionts is a major influence on systemic biological processes within an organism^[5]^. Previous studies have determined that endosymbionts influence fundamental aspects of host insect biology, including nutritional mechanisms^[6]^, reproduction^[7]^, tolerance of abiotic conditions^[8,9]^, and the reduction of an insect’s ability to vector pathogens^[10]^. Molecular pathways critical to the health of endosymbionts and insect hosts provide targets of interest for designing therapeutics to induce phenotypic changes in the insect. Additionally, symbiotic relationships are of interest in evolutionary genetics, as codependence can result in genetic markers in endosymbiont genomes, which inform the evolutionary history and adaptation of the insect^[11,12]^. Insect diets deficient in vitamins, sterols, and essential amino acids^[13]^ are supplemented by symbiotic microorganisms that produce the necessary dietary nutrients^[14]^. Sap ingesting psyllids, such as *L. fagarae*, are hosts to a variety of bacterial endosymbionts, most notably Gram-negative *Wolbachia* of the order Rickettsiales, which are currently found in 40%-60% of insect species^[15,16]^. Our characterization of *Wolbachia* in *L. fagarae* is specifically noteworthy due to *Wolbachia*’s potential to modulate the fitness of disease-vectoring insects^[17]^ and its close relationship to the *Wolbachia* strain found in the Huanglongbing (HLB) vector *Diaphorina citri*. Previous analysis of *Wolbachia* genome assemblies further determined that these microorganisms are without complete sets of metabolic pathways present in closely related, nonsymbiotic bacteria of the order *Rickettsiales*^[18]^, suggesting that *Wolbachia* can manipulate host metabolic pathways^[19,20]^. This dynamic influence, along with the exclusive vertical transmission through maternal hosts, contributes to the exploitation of major reproductive influences through *Wolbachia*-host relationships^[21]^.

*Wolbachia* are currently classified into separate monophyletic lineages, or supergroups, denoted A to S^[22]^. Here, we looked to classify the evolutionary placement of *Wolbachia* endosymbiont of *Leuronota fagarae* (wLfag-FL) in the supergroup classification system and to examine the transcriptome of wLfag-FL to uncover conserved or unique transcripts informing the symbiotic relationships of *Wolbachia* and psyllid hosts. Our study uses an multilocus sequence typing (MLST) of nine loci to classify wLfag-FL phylogeny and leverages published *Wolbachia* genomes from *D. citri* to perform a comparative analysis of *Wolbachia* loci found in two psyllid species invasive to Florida, USA. *L. fagarae* presents an important organism to study in parallel to *D. citri*, as recent research on the Australian finger lime produced a novel line of stable antimicrobial peptides (SAMPs) to bolster citrus immunity against CLas infection. Since *L. fagarae* feeds on the citrus relative wild lime, is closely related to *D. citri*, and is invasive to Florida, the biology of *L. fagarae* and its endosymbionts informs HLB research and may reveal more naturally occurring therapeutic molecules to combat HLB. A thorough comparative investigation of the wLfag-FL transcriptome was not possible due to the low number of transcripts recovered from wLfag-FL, although transcripts encoding proteins essential for nutrient provisioning, proteins with secretory domains, and proteins with ankyrin repeat (ANK) domains characteristic to *Wolbachia* were identified to inform host-microbe interactions.

## METHODS

### *L. fagarae* collection

Colonies of wild-caught *L. fagarae* found on *Zanthoxylum fagara* were maintained at the University of Florida Southwest Florida Research & Education Center in Immokalee, FL, U.S.A., for 5 years. *L. fagarae* colonies were maintained on citrus relative *Zanthoxylum fagara* (L.) Sarg (Sapindales: Rutaceae) by the Qureshi lab and sample collection from the colony occurred in 2017. After collection, psyllids were immediately placed into TRIzol^TM^-LS Reagent (Invitrogen^TM^ #10296028, 200 mL) for RNA purification^[23]^.

### RNA extraction, cDNA preparation, and library sequencing

Four cDNA libraries were made from RNA extracted from salivary glands, heads, or whole-body tissue of *L. fagarae*. Both cDNA libraries from the salivary gland and head tissues were obtained from combined male and female samples, while both whole-body cDNA libraries were made from either male or female psyllids. In total, 900 psyllids were collected to build cDNA datasets where 500 psyllids were dissected for the mixed salivary gland dataset, 380 psyllids were dissected for the mixed heads dataset, and 10 psyllids each, male and female, were used for the whole-body dataset RNA extractions. RNA was purified using the Direct-zol RNA MicroPrep method for the adult whole body and mixed heads extractions, and TRIzol LS, Na-Acetate/ethanol precipitation was used to purify the mixed salivary glands extractions. Total RNA concentration dissolved in nuclease-free water was assessed with the Qubit BR assay. The results showed that the mixed salivary gland extractions yielded 2.8 ng/μL of RNA, the mixed head extractions yielded 4.4 ng/μL of RNA, adult males yielded 30.8 ng/μL of RNA, and adult females yielded 134 ng/μL of RNA. To sequence the transcriptome of each tissue or whole psyllid, we used the NEB Ultra II Directional RNAseq Library Prep Kit from Illumina paired with the Illumina NextSeq 500 Mid mode throughput 2X150 method. Adapters and low-quality reads (Q < 20) were removed at the University of Florida Interdisciplinary Center for Biotechnology Research (UF|ICBR). Raw reads have been deposited into the Sequence Read Archive under BioProject PRJNA789049 and assembled transcripts are found in BioSample SAMN46999246.

### Data processing and sequence identification

De novo assemblies of four resulting cDNA libraries (salivary glands, heads, whole-body males, and whole-body females) were performed using the TRINITY RNA-seq assembler v1.8.0^[24]^ with default parameters. For the initial characterization of *Wolbachia* sequences, nine local databases containing conserved *Wolbachia* housekeeping genes: 16S rRNA, *wsp*, *coxA*, *fbpA*, *ftsZ*, *gatB*, *gltA*, *groEL*, and *hcpA* were built from transcripts available on NCBI. The Trinity assembled transcripts were aligned to each of the nine local databases using BLASTn. The longest transcripts matching a subject with the lowest E-value scores were selected for MLST. Phylogenetic analysis was performed using a concatenated form of these conserved sequences for each of the samples listed in Table 1. As a result of the MLST study, the three most closely related *Wolbachia* endosymbionts to wLfag-FL: *Spodoptera picta* strain Spic_B (CP067976.1), *Diaphorina citri* strain dawsonii (CP051608.1), and *Laodelphax striatellus* strain wStri (NZ_MUIX01000001.1) were used as reference genomes for HISAT2 v2.0.4 mapping with default parameters^[25]^ to identify *Wolbachia* sequences in the four TRINITY assembled datasets. The resulting sequences were subject to BLASTn analysis using the NCBI nt database to verify sequence identity across a broader database. If sequences did not find a match to NCBI via nucleotide, BLASTx was used instead to compare protein homology. BUSCO v5.2.2 was used to assess the transcriptome completeness of obtained *Wolbachia* orthologs^[26]^. The CAP3 long read assembler in UGENE v40^[27]^ was used to further assemble fragmented *Wolbachia* sequences. The contigs that did not assemble any further were grouped with the CAP3 contig assemblies, which were subject to gene annotation.

**Table 1:** List of all *Wolbachia* strains and accessions used in phylogenetic analysis

**Host name**	**Common name**	**Taxonomy (order: family)**	**Strain**	**Supergroup**	**Abbreviation**	**GenBank accession**
*Anastrepha fraterculus*	South American fruit fly	Diptera: Tephritidae	clone 46.1	A	wAfra†	KC589027.1
*Carposina sasakii*	Peach fruit moth	Lepidoptera: Carposinidae	*w*CauA	A	*w*Csas	NZ_CP041215.1
*Ceratosolen solmsi*	Fig wasp	Hymenoptera: Agaonidae	MIAOwCsol	A	*w*Csol	CP054598.1
*Drosophila ananassae*	Fruit fly	Diptera: Drosophilidae	*w*Ana	A	*w*Dana	CP042904.1
*Drosophila incompta*	Fruit fly	Diptera: Drosophilidae	*w*Inc Cu	A	*w*Dinc	NZ_CP011148.1
*Drosophila melanogaster*	Fruit fly	Diptera: Drosophilidae	*w*Melpop	A	*w*Dmel	CP046921.1
*Drosophila simulans*	Fruit fly	Diptera: Drosophilidae	*w*Au	A	*w*Dsim1	CP069055.1
*Hypothenemus hampei*	Coffee berry borer	Coleoptera: Curculionidae	H control 1	A	wHham†	KX436087.1
*Wiebesia pumilae*	Fig wasp	Hymenoptera: Agaonidae	MIAOwWpum	A	*w*Wpum	CP054557.1
*Aedes albopictus*	Tiger mosquito	Diptera: Culicidae	*w*AlbB	B	*w*Aalb	CP031221.1
*Bactericera cockerelli*	Potato psyllid	Hemiptera: Triozidae	clone 3	B	wBcoc†	KM267307.1
*Bemisia tabaci*	Silverleaf whitefly	Hemiptera: Aleyrodidae	China 1	B	*w*Btab	NZ_CP016430.1
*Culex quinquefasciatus*	Southern house mosquito	Diptera: Culicidae	*w*PipPel	B	*w*Cqui	AM999887.1
*Diaphorina citri*	Asian citrus psyllid	Hemiptera: Liviidae	dawsonii	B	*w*Dcit1	CP051608.1
*Diaphorina citri*	Asian citrus psyllid	Hemiptera: Liviidae	KPSwDI10P38	B	*w*Dcit2	CP051265.2
*Diaphorina citri*	Asian citrus psyllid	Hemiptera: Liviidae	Beihai	B	*w*Dcit3†	GQ385974.1
*Diaphorina citri*	Asian citrus psyllid	Hemiptera: Liviidae	Shenzhen	B	*w*Dcit4†	GU480072.1
*Drosophila mauritiana*	Fruit fly	Diptera: Drosophilidae	*w*Mau	B	*w*Dmau	CP034335.1
*Drosophila simulans*	Fruit fly	Diptera: Drosophilidae	*w*Ma	B	*w*Dsim2	CP069054.1
*Homalodisca coagulata*	Glassy-winged sharpshooter	Hemiptera: Cicadellidae	GWSS	B	*w*Hcoa†	DQ450164.1
*Homalodisca elongata*	Glassy-winged sharpshooter	Hemiptera: Cicadellidae	B21HE	B	*w*Helo†	DQ450154.1
*Laodelphax striatellus*	Small brown planthopper	Hemiptera: Delphacidae	*w*Stri	B	*w*Lstr	NZ_MUIX01000001.1
*Nilaparvata lugens*	Brown planthopper	Hemiptera: Delphacidae	*w*Lug	B	*w*Nlug	NZ_MUIY01000001.1
*Spodoptera picta*	Lily caterpillar	Lepidoptera: Noctuidae	Spic B	B	*w*Spic	CP067976.1
*Brugia pahangi*	Filarial nematode worm	Rhabditida: Onchocercidae	FR3	D	*w*Bpah	CP050521.1
*Litomosoides sigmodontis*	Filarial nematode worm	Rhabditida: Onchocercidae	*w*Lsig	D	*w*Lsig	CP046577.1
*Folsomia candida*	Springtail	Collembola: Isotomidae	Berlin	E	*w*Fcan	NZ_CP015510.2
*Cimex lectularius*	Bed bug	Hemiptera: Cimicidae	*w*Cle	F	*w*Clec	AP013028.1

†: *Wolbachia* strains included only in the ML phylogenetic tree depicting evolutionary lineage by the *wsp* gene.

### Phylogenetic characterization

Two methods were employed to characterize the evolutionary lineage of *Wolbachia* found in *L. fagarae* from Florida. The first approach used a concatenated sequence of nine conserved genes, where sequences were only gathered and analyzed if they were part of a fully assembled *Wolbachia* genome on NCBI. The resulting concatenated sequence alignment, performed with MUSCLE in UGENE v40, totaled 7,655 nucleotides in length, where partial sequences of *CoxA*, *ftsZ*, *gatB*, *gltA*, *groEL*, and *hcpA* were used along with full-length sequences of *fbpA*, *wsp*, and 16S rRNA. The second approach used a portion of the *wsp* gene that encodes a transmembrane structure, which plays a role in controlled cell death, cell proliferation, pathogenicity, and host immune response^[28]^. The *wsp* sequences obtained from NCBI, belonging to various *Wolbachia* strains of interest, were aligned with MUSCLE in UGENE v40, and trimmed to the limiting sequence, resulting in a length of 609 nucleotides. Both phylogenetic trees were built using a bootstrap value of 1000 and the default Maximum Likelihood parameters in UGENE v40.

### Transcriptome functional annotation

Four *Wolbachia* gene datasets available in the KEGG Automatic Annotation Server (KAAS v2.1) were used to obtain KEGG Orthology identifiers of wLfag-FL sequences^[29-31]^. The four datasets added together totaled 4,425 sequences and were entitled *Wolbachia* endosymbiont of *Drosophila melanogaster* (wol), *Wolbachia* sp. wRi (wri), *Wolbachia* endosymbiont of Culex quinquefasciatus Pel (wpi), and *Wolbachia* endosymbiont strain TRS of Brugia malayi (wbm). Both unassembled and CAP3 assembled sequences were placed into the KAAS server for annotation via the BLAST method and SBH approach. Identification of sequences containing ankyrin domains was carried out post CAP3 assembly of identified wLfag-FL contigs and was initiated by BLASTx analysis to a dataset of 182 Multispecies *Wolbachia* ankyrin domain-containing proteins from NCBI. Aligned sequences were then verified by selecting whole ExPASy sequence translations^[32]^ of the same frames, which had homology to *Wolbachia* ANK domain proteins predicted by BLASTx. The translated sequences were compared against the UniProt database for final confirmation.

## RESULTS

### Phylogenetic analysis

**Figure 1:**
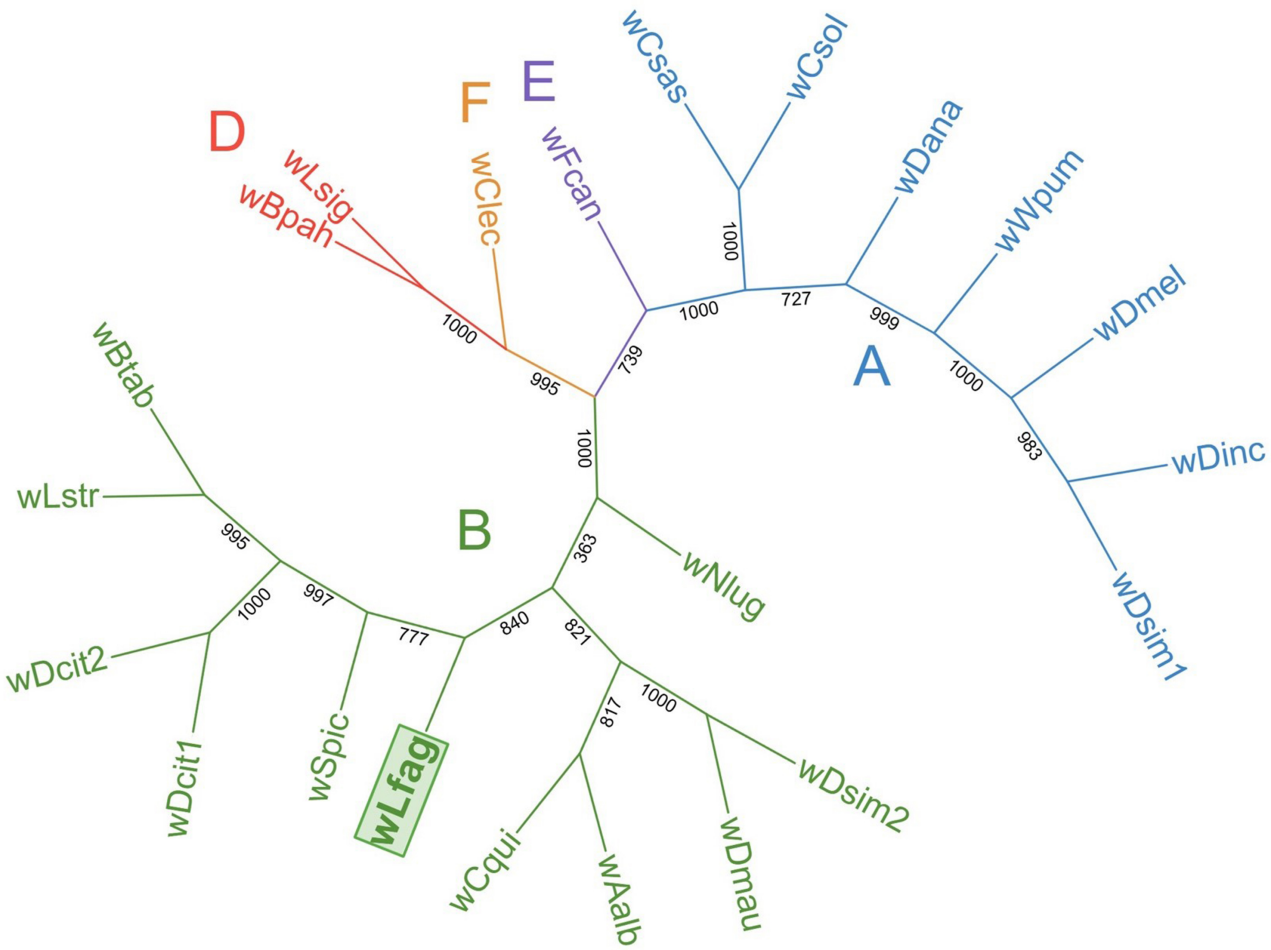
Unrooted Maximum Likelihood tree for *Wolbachia* MLST concatenated sequences supporting placement of wLfag-FL in Supergroup B. MLST: Multilocus sequence typing; wLfag-FL: *Wolbachia* endosymbiont of *Leuronota fagarae.*

**Figure 2:**
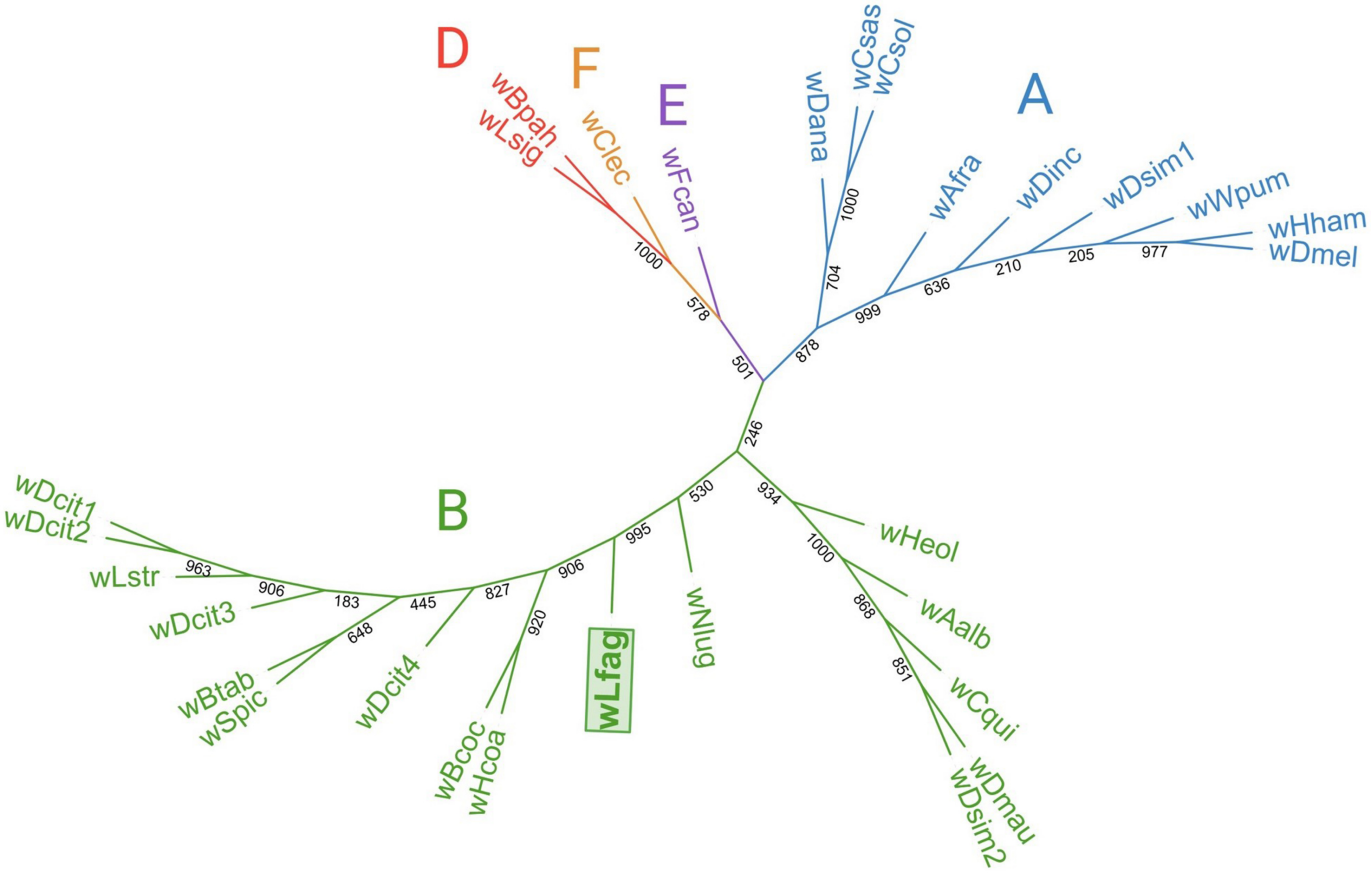
Unrooted Maximum Likelihood tree for *Wolbachia wsp* sequences highlighting the placement of wLfag-FL in Supergroup B with a distinct evolutionary lineage. wLfag-FL: *Wolbachia* endosymbiont of *Leuronota fagarae.*

Both phylogenetic characterization strategies using MLST [Figure 1] and *wsp* [Figure 2] assigned *Wolbachia* strain wLfag-FL to supergroup B, which contains closely related psyllids along with other Hemipterans, Dipterans, and a Lepidopteran. A *wsp* gene coding for a surface protein of *Wolbachia* was specifically chosen for further analysis as it evolves at a much faster rate than other genes such as *16S* or *ftsZ*, and has been previously used in characterizing *Wolbachia* strains^[33]^. Moreover, the *wsp* gene is allotted for further analysis of strains without a completely assembled genome. Analysis was attempted with a protein translation of the *wsp* gene, but given the lack of whole sequence availability for other *Wolbachia* strains, incomplete protein translations could not differentiate lineages of closely related strains. The *Wolbachia* endosymbiont of *B. cockerelli* is seemingly the closest relative of wLfag-FL according to *wsp* analysis. All sequence information used to build the phylograms is recorded in Table 1. A depiction of the wsp alignment created in Jalview v2.11.1^[34]^ is available in Supplementary Figure 1.

### Transcriptome analysis

**Figure 3:**
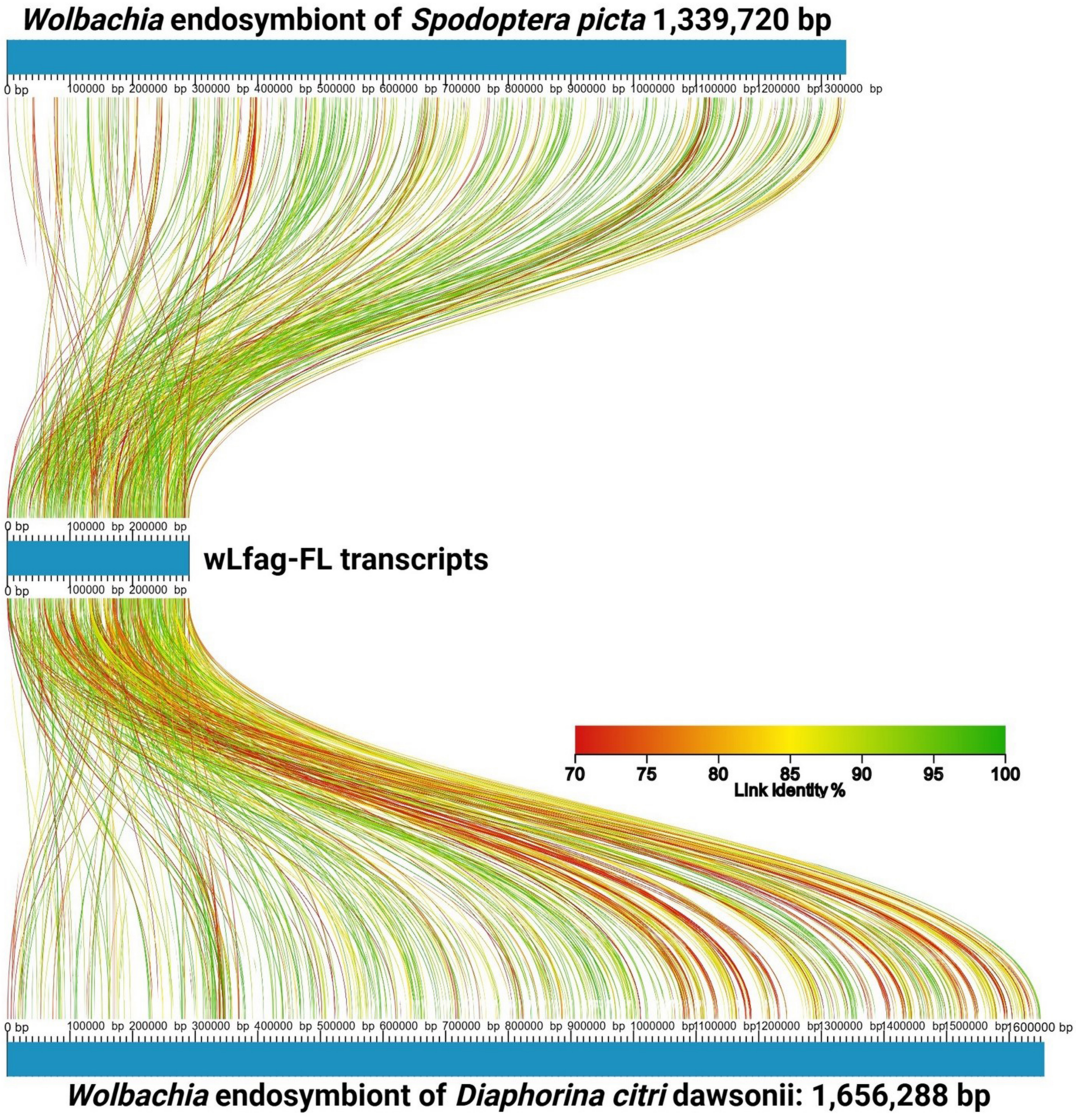
AliTV linear alignment map of wLfag-FL concatenated transcripts mapped to closely related *Wolbachia* genomes of wDcit1 and wSpic. Accession numbers of full genome references are in Table 1. wLfag-FL: *Wolbachia* endosymbiont of *Leuronota fagarae;* wDcit1: *Wolbachia* endosymbiont of *D. citri* dawsonii; wSpic: *Wolbachia* endosymbiont of *S. picta.*

**Figure 4:**
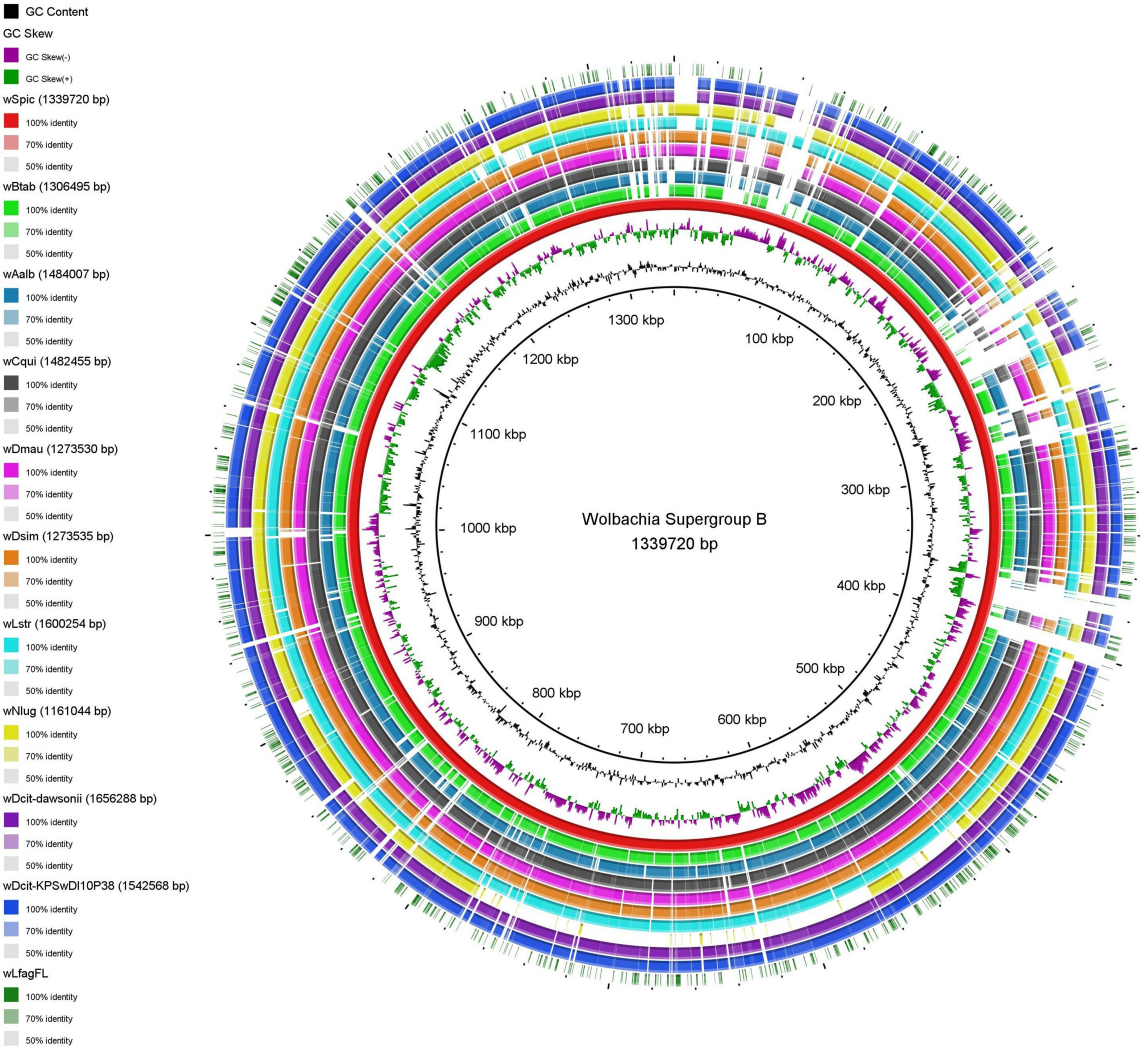
BRIG alignment of 11 *Wolbachia* endosymbiont genomes belonging to Supergroup B used in the MLST phylogenetic classification of wLfag-FL. *Wolbachia* endosymbiont of *S. picta* was set as the reference genome (red); the outermost ring (dark green) is made of wLfag-FL transcripts. Genome assembly lengths are in parenthesis next to the names of the *Wolbachia* strain. Genome accessions are in Table 1. BRIG: Blast Ring Image Generator; MLST: multilocus sequence typing; wLfag-FL: *Wolbachia* endosymbiont of *Leuronota fagarae.*

Of the 1,359 transcripts mapped to reference *Wolbachia* genomes by HISAT2, only six sequences did not match *Wolbachia* through BLASTn to NCBI *nt*; however, these 6 did match *Wolbachia* sequences through BLASTx to NCBI *nr* with the greatest E-value reported as 4e-08. To assess transcriptome completeness of the assembled *Wolbachia* cDNA sequences, Benchmark Universal Single Copy Ortholog (BUSCO) v5.2.2 was used^[26]^. In total, 1,332 non-redundant transcripts were compared against the rickettsiales_odb10 database, using transcriptome mode. BUSCO calculated only 37 Complete BUSCOs (10.7%), 14 Fragmented BUSCOs (4.1%), and 294 Missing BUSCOs (85.2%). Seqkit v0.16.1^[35]^ was used to calculate a GC content of 34.8% and an N50 value of 348 nt for Trinity assembled *Wolbachia* transcripts. When aligning the wLfag-FL transcripts to the annotated sequences of *w*Dcit dawsonii and the *w*Spic, we identified 634 and 560 alignments to unique transcripts, respectively, and revealed, on average, a 45% annotation rate of our wLfag-FL Trinity transcripts. Roughly 2.5 times more *Wolbachia* sequences were recovered from the female *L. fagarae* dataset compared to orthologs recovered in the male dataset. To visualize sequence comparisons between wLfag-FL and other closely related *Wolbachia* strains, we used AliTv^[36]^ and the Blast Ring Image Generator (BRIG)^[37]^. An AliTv alignment mapping figure was generated with concatenated wLfag-FL transcripts, post CAP3 assembly, and *Wolbachia* reference genomes *Spodoptera picta* Spic B and *Diaphorina citri dawsonii* [Figure 3]. To generate the BRIG image [Figure 4], Supergroup B *Wolbachia* genomes from the MLST phylogeny were reused.

### Functional annotation

**Figure 5:**
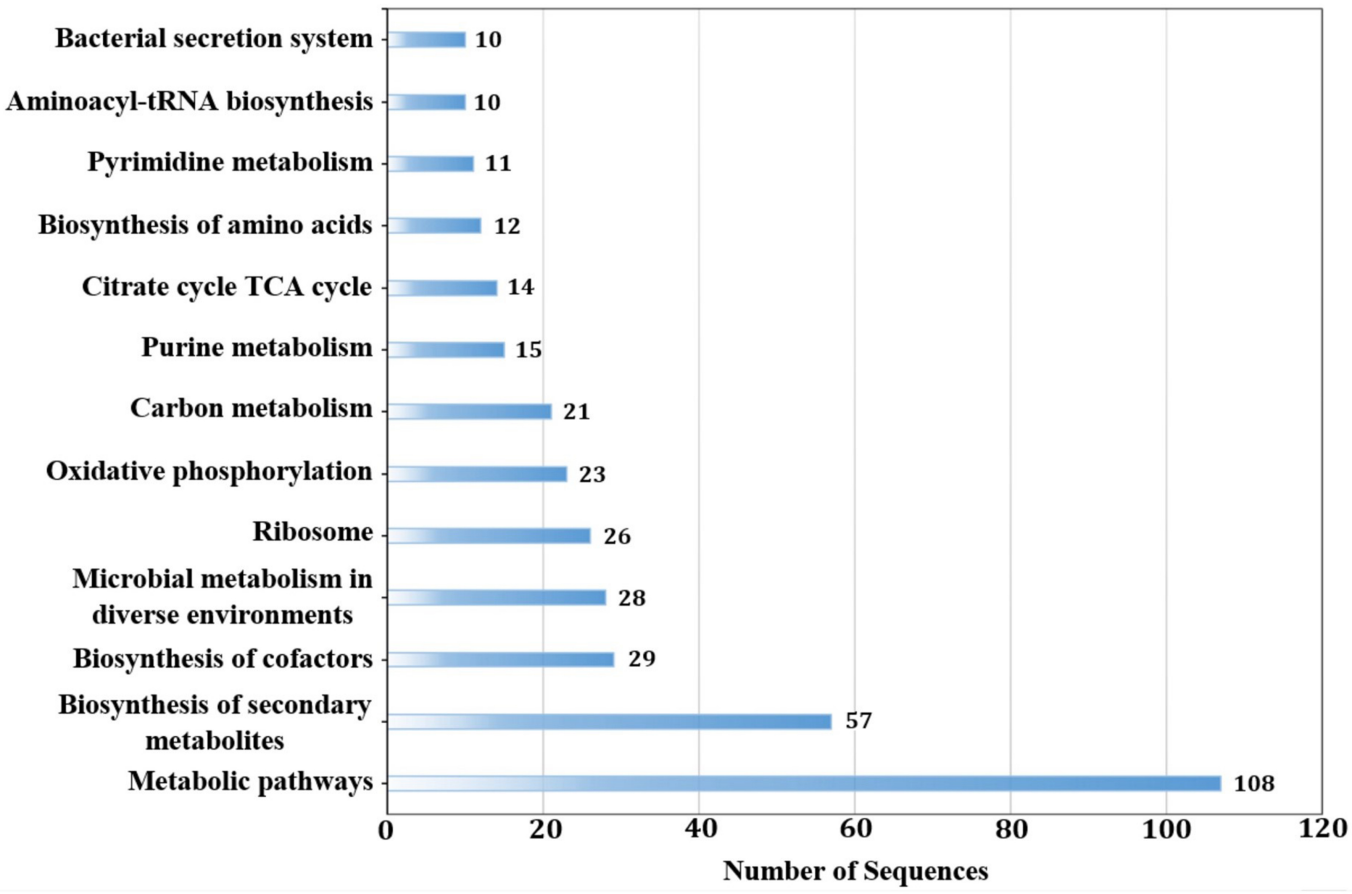
Bar graph containing 13 KEGG Orthology pathways having 10 or more sequence assignments from *Wolbachia* identified in *L. fagarae* FL-isolates. A full table of sequences assigned to KO identifiers is available in Supplementary Table 1.

As a result of the KEGG ontology analysis, 465 transcripts were assigned a K number, of which 279 were unique of the 828 total sequences examined. The map pathway function available in the KO Database was used to group the unique K number assignments into their associated pathways. The KEGG Orthology Metabolic pathway (ko01100) contained the highest number of assignments with 108 sequences. The bar graph [Figure 5] depicts an abundance of 13 KEGG Orthology pathways containing at least 10 sequence assignments. Supplementary Table 1 contains a complete list of K numbers from all unique wLfag-FL sequences assigned to 146 pathways, as well as their assigned sequence names. Analysis of ANK domain-containing proteins performed on cDNA sequences of wLfag-FL revealed five sequences containing ankyrin domains. This was expected though, as BUSCO analysis revealed only 10.7% of the endosymbiont’s transcriptome was recovered from the four datasets, and that other closely related *Wolbachia* strains of Supergroup B contain around 50-60 ANK domain proteins. Our identification of 5 ANK domain-containing proteins is consistent with our BUSCO score and average number of ANK domain proteins in Supergroup B strains which predicts that wLfag-FL encodes roughly 50 ANK domain proteins.

**Figure 6:**
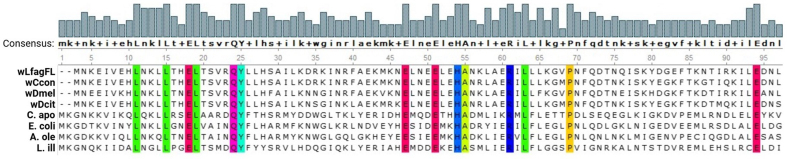
MUSCLE alignment of 8 bacterioferritin sequences. Residues with a 100% identity between all 8 sequences are highlighted. Sequence accessions are available in Supplementary Table 2.

The reproductive advantages provided by *Wolbachia*-host symbiosis are critical aspects of the symbiotic relationship; however, *Wolbachia* provides another method to increase host fitness by supporting nutritional provisioning. A 5’ portion of the *Wolbachia* bacterioferritin CDS was present in wLfag-FL, having the closest similarity to bacterioferritin characterized in the *Wolbachia* endosymbiont of *Homalodisca vitripennis* (MBR9983968.1). To verify the identity of the partial bacterioferritin transcript, we used ExPASy translate to identify the correct protein sequence and aligned previously characterized bacterioferritin protein sequences, discovered by methods other than bioinformatic analysis, with MUSCLE. We found the highly conserved residues within the ferroxidase center to align between all 8 sequences [Figure 6]. Supplementary Table 2 contains accession numbers of sequences used in the bacterioferritin protein alignment. Furthermore, direct comparisons of the wLfag-FL transcriptome to full and partially sequenced *Wolbachia* genomes from *Diaphorina citri* hosts identified protein domains of phytanoyl-CoA dioxygenase and collagen-like proteins associated with metabolic processes in wLfag-FL and other *Wolbachia* strains, which are seemingly absent in wDcit sequences.

Proteins secreted by a disease vector are of significance on account of their central role within a broad range of processes through which an organism can elicit infection and evoke intercellular communication. Of all sequences identified by KEGG as belonging to the secretion system pathways, two were affiliated with Type I secretion (T1SS), four with Type IV secretion (T4SS), and five with the Sec-pathway where one of the five was identified as the signal recognition particle (SRP) protein encoded by the *ffh* gene, which was identified as the prokaryotic counterpart of the eukaryotic 54 kDa subunit of SRP (SRP54). The other Sec-pathway components identified were secA, an ATPase, as well as secD, secE, and secF, which are all integral membrane proteins. Gram-negative bacteria T1SS outer membrane protein TolC was identified along with a T1SS system ATPase. T4SS proteins VirB4, VirB6, VirB9, and VirB11 were additionally identified.

## DISCUSSION

*Wolbachia* is a maternally inherited bacterium pervasively found in insect species^[38]^. The number of studies surrounding *Wolbachia* has increased due to an expanded understanding of the microorganism’s influence on reproductive systems, fitness, and development traits in its host. In some insects, *Wolbachia* are known to upregulate egg production in maternal hosts^[18,21,39]^, and increase recombination of the host X chromosome, resulting in proportionately high genetic divergence^[40]^. Our study presents the first characterization of *Wolbachia* in *L. fagarae* and our results from MLST and *wsp* analysis indicate we have recovered transcripts from a new strain. Therefore, based on prior classification per the established nomenclature system^[41]^, we propose the designation wLfag-FL as the species strain ID of the *Wolbachia* isolate from wild lime psyllids in the state of Florida, United States.

To identify the strain of *Wolbachia*, multiple housekeeping genes circulated over the genome were used as a core marker gene set, which is suggested for accurate *Wolbachia* strain genotyping^[42]^. The *wsp* gene sequence was further utilized to depict relations to other *Wolbachia* strains without full genome assemblies. In both analyses, wLfag-FL consistently grouped with *Wolbachia* from Supergroup B, which contains other closely related psyllid species known to house disease-causing Liberibacteria. We also found that wLfag-FL is closely related to the *Wolbachia* resident of *B.* cockerelli, further supporting that both insects originated from Central America. One other important insect pest of South and Central America is the coffee berry borer *Hypothenemus hampei*, which is credited for causing losses of more than a half billion USD in the coffee-producing industry^[43]^. *H. hampei* origins are traced to tropical biomes of Africa and its *Wolbachia* endosymbiont grouped with Drosophila melanogaster, another insect of African origin. The placement of other *Wolbachia* strains into the correct supergroups strengthens the interpretation of our MLST analysis.

Regarding endosymbiotic *Wolbachia*, one of the most intriguing components for host interaction is the substantial number of genes encoding proteins with ANK domains. As few as five ANK domain-containing proteins were identified in the *Wolbachia* endosymbiont of *Brugia malayi*, and as many as 60 were identified in the Wolb-pip strain of *Wolbachia* from the *Culex pipiens* group^[44]^. Annotation of the *Wolbachia* endosymbiont of the *Diaphorina citri* genome revealed 54 predicted proteins containing ANK domains^[45]^. *ANK* genes in *Wolbachia* have been proposed as major factors in the endosymbiont’s functional role and unique symbiotic relationship. Functions of ANK-containing proteins acting as binding molecules in *Wolbachia* are difficult to predict since few have sequence similarity outside the ankyrin domains to other proteins of known functions^[46]^. Nevertheless, many studies suggest that *Wolbachia* ankyrin domain proteins are involved in many symbiotic pathways, expanding the understanding of how intertwined *Wolbachia*-host interaction truly is. Biological processes such as the assembly of cation channels, transcriptional regulation, cell differentiation, apoptosis, cell signaling, and most importantly, secretion systems are all thought to contain some sort of protein embodying the ANK motif^[47-49]^. Further analysis of ANK domain proteins of wLfag-FL must be conducted to obtain a clearer understanding of their roles in this specific *Wolbachia*-host symbiosis, as our recovery of only 5 transcripts is not enough to draw extensive conclusions.

By influencing host metabolic processes, *Wolbachia* enhance their selection process as the reproductive assistance they provide may not benefit all hosts selected for infection. Provided the extensive gene loss in endosymbiotic *Wolbachia*, it is inferred that these strains are nutritionally dependent upon their hosts. Bacterioferritin, the bacterial homolog of eukaryotic ferritin, has been inferred to help eukaryotic hosts capture excess Fe(2+) to avoid iron toxicity and allow the ferric mineral to have increased bioavailability^[50]^. The three coordination environments (A, B, C) in the ferroxidase center of bacterioferritin are highly conserved, consisting of four alpha helices (A, B, C, and D). Helix A is characterized by two highly conserved αα’s Glu18, and Tyr25, Helix B by αα’s Glu47, Glu51, and His54, Helix C by αα Glu94, and Helix D by αα’s Asp126, Glu127, Gly129, and His130^[51]^. All the listed conserved residues were shown to exist in wLfag-FL bacterioferritin, except the residues of Helix D, as the translated protein sequence obtained from wLfag-FL accounts only for the first 95 residues of the *Wolbachia* bacterioferritin protein sequence.

Other predicted metabolites and transporters have been identified in wLfag-FL, inferring the provision of biological molecules for the metabolism of riboflavin, glutathione, glucose, carbon, pentose phosphates, pyrimidines, purines, fatty acids, and various amino acids. Transporters associated with various substrates including pyruvate, carbohydrates, amino acid catabolism, and inorganic cations were additionally identified^[46,52,53]^. Analysis of the wLfag-FL transcriptome identified a sequence encoding a phytanoyl-CoA dioxygenase family protein, which catalyzes conversions of alpha-ketoglutarate to succinate, and phytanoyl-CoA to 2-hydroxyphytanoyl-CoA^[54]^. The generation of succinate by phytanoyl-CoA dioxygenase, a family member of iron(II)-dependent oxygenases, has been suggested to aid in energy production under conditions of nutritional stress^[55,56]^. It has been proposed that in predicted operons combining 2OGFeDOs of phytanoyl CoA oxygenase with sulfotransferases, there are collagen-like domain proteins thought to act as substrates of the related co-encoded enzymes^[57]^. A collagen-like protein coding sequence, containing 20 copies of a collagen triple helix repeat identified by Pfam v34.0^[58]^, was found in wLfag-FL and has homology to another collagen-like protein identified in *Wolbachia* endosymbionts containing the beforementioned phytanoyl-CoA dioxygenase family protein. However, this specific collagen-like protein had no homology to collagen-like proteins identified in wDcit endosymbionts, suggesting collaboration with a hydroxylation system no longer present in the *Wolbachia* resident of *Diaphorina citri*. Other protein coding regions of interest involved in various processes were identified in wLfag-FL that had no homology to coding regions within wDcit genome assemblies. These sequences encoded for S-adenosylmethionine uptake transporter, UDP-glucose 6-dehydrogenase, and an IS66 family transposase. S-adenosylmethionine uptake transporter of the DMT-superfamily contributes to the transport of S-adenosylmethionine required by various biological pathways. UDP-glucose 6-dehydrogenase is associated with outer cell surface glycostructures such as lipopolysaccharide biosynthesis. IS66 family transposase is necessary for DNA transposition and may cause structural variations in bacterial plasmids. Further research is necessary to identify other gene products involved with the three mentioned proteins, as around 10% of the transcriptome was recovered. Enrichment for this new *Wolbachia* strain and deeper sequencing must be conducted to detect other genetic elements, enabling full transcriptome or genome comparisons. As *Wolbachia* exhibit important roles with an insect’s microbiome, identification of genes necessary to uphold the symbiotic relationship may provide targets for RNAi.

Fundamental pathways used to assess the combativeness of an infection are those containing secreted proteins. KEGG analysis revealed proteins from the T1SS, T4SS, and Sec pathways in wLfag-FL. From the Sec-pathway, SRP *ffh* was identified and has been shown to work in cohorts with a ribosome-associated chaperone and peptidyl-prolyl isomerase, which determine whether an actively synthesized protein will be inserted into the outer membrane or exported through T2SS^[59]^. Provided the conservation of the *ffh* coding region between *Wolbachia* strains, it may serve as a component in future MLST studies to assist in the further classification of newly sequenced *Wolbachia* strains. The T4SS system is one of several types of secretion systems utilized by microorganisms for the transport of macromolecules across the cell envelope, and disruption of this pathway, preventing protein oligomerization, may invoke lower infection rates of various bacterial species. Lastly, the identification of T4SS *Vir* proteins suggests that both operons within the virB-virD4 loci are present in wLfag-FL^[60]^.

### Limitations

Recovery of the complete *Wolbachia* transcriptome from the four cDNA datasets was not successful, as determined by our low BUSCO scores and low percentage of unique transcripts mapped to transcripts of related *Wolbachia*. We believe this low number of complete transcripts is due to the fragmented nature of the transcript assembly and the inability of BUSCO to identify suitable protein alignments. Our N50 value is relatively low compared to N50s of 1,215 for wDcit dawsonii and 1,236 for wSpic. We attempted to further assemble the transcripts using CAP3, which negligibly increased our N50 to 751; however, this resulted in a lower complete BUSCO score. Figures 3 and 4 show a complete distribution of transcripts throughout whole reference genomes, and not just 15% of the genomes, which would be expected considering our BUSCO score. Short transcripts, around 150-250 bp, still mapped regularly throughout entire reference genomes, suggesting their presence, although complete annotation was not possible through BUSCO or KEGG analysis. This assessment was not surprising as *Wolbachia* was not the target of initial sequencing efforts and, therefore, sequencing of nucleic acids enriched for microorganisms by a pretreatment DNase and filtration protocol^[61]^ must be performed for a complete assessment of the wLfag-FL transcriptome. Furthermore, long-read sequencing of the genomic DNA will reveal a more complete assessment of the total CDS encoded in the wLfag genome.

In conclusion, The comprehensive screening and classification of *Wolbachia* in *L. fagarae* has broadened the host spectrum of *Wolbachia* identified in members of Hemiptera: Psylloidea. A detailed MLST classification of conserved housekeeping genes is standard practice and an effective method to determine the phylogenetic classification of genetically divergent *Wolbachia* strains. The newly identified *Wolbachia* strain in *L. fagarae* provides conserved biological elements that may be manipulated to influence host vector fitness and fecundity. This study allows for the advancement of biological control applications that do not involve the use of environmentally harmful chemicals, thus focusing on environmentally safer management strategies for pest control while protecting the ecosystem.

## DECLARATIONS

### Availability of data and materials

The data supporting this work is provided in the text. Additional raw data will be made available by the corresponding author upon reasonable request.

### Copyright

© The Author(s) 2025.
