# Supplementary material for: Transcriptomic characterization of Wolbachia endosymbiont from Leuronota fagarae (Hemiptera: Psylloidae)
Source: Microbiome Res Rep. 2025 Apr 3;4(2):19. doi: 10.20517/mrr.2024.84 (PMC12369390; doi:10.20517/mrr.2024.84)
Supplement: Supplementary file 1 [file mrr-4-2-19-SupplementaryMaterials.pdf]

Supplementary Materials

Transcriptomic characterization of *Wolbachia* endosymbiont from *Leuronota fagarae* (Hemiptera: Psylloidae)

Douglas S. Stuehler Jr.[1,2], Wayne B. Hunter[2], Jawwad A. Qureshi[3], Liliana M. Cano[4]

[1]Oak Ridge Institute for Science and Education ORISE, Department of Energy and the United States Department of Agriculture DOE/USDA, Agricultural Research Station ARS, Fort Pierce, FL 34945, USA.
[2]United States Department of Agriculture USDA, Agricultural Research Station ARS, U.S. Horticultural Research Laboratory, Fort Pierce, FL 34945, USA.
[3]Department of Entomology and Nematology, Southwest Florida Research and Education Center SWFREC, Institute of Food and Agricultural Sciences IFAS, University of Florida, Immokalee, FL 34142, USA.
[4]Department of Plant Pathology, Indian River Research and Education Center IRREC, Institute of Food and Agricultural Sciences IFAS, University of Florida, Fort Pierce, FL 34945, USA.

Correspondence to: Dr. Liliana M. Cano, Department of Plant Pathology, Indian River Research and Education Center IRREC, Institute of Food and Agricultural Sciences IFAS, University of Florida, 2199 South Rock Road, Fort Pierce, FL 34945, USA. E-mail: Lmcano@ufl.edu

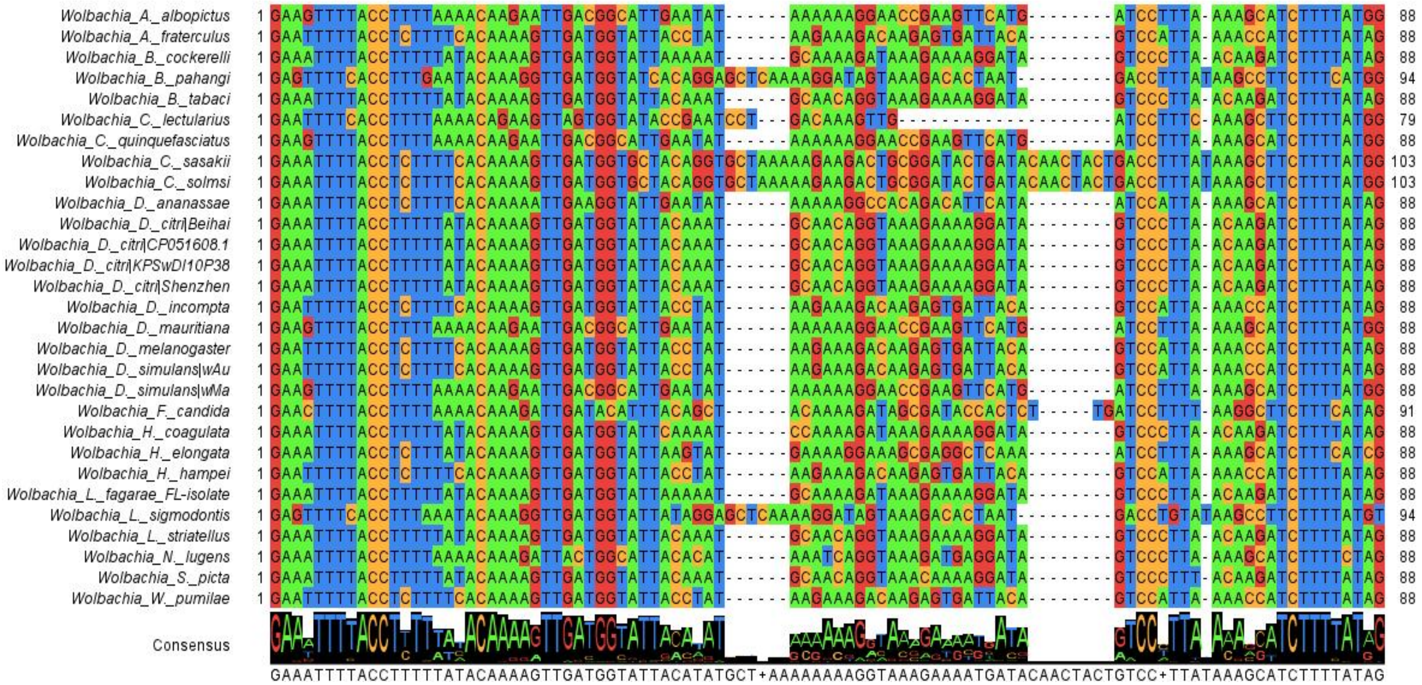
Wolbachia_A._albopictus
Wolbachia_A._fraterculus
Wolbachia_B._cockerelli
Wolbachia_B._pahangi
Wolbachia_B._tabaci
Wolbachia_C._lectularius
Wolbachia_C._quinquefasciatus
Wolbachia_C._sasakii
Wolbachia_C._solmsi
Wolbachia_D._ananassae
Wolbachia_D._citri|Beihai
Wolbachia_D._citri|CP051608.1
Wolbachia_D._citri|KPSwDI10P38
Wolbachia_D._citri|Shenzhen
Wolbachia_D._incompta
Wolbachia_D._mauritiana
Wolbachia_D._melanogaster
Wolbachia_D._simulans|wAu
Wolbachia_D._simulans|wMa
Wolbachia_F._candida
Wolbachia_H._coagulata
Wolbachia_H._elongata
Wolbachia_H._hampei
Wolbachia_L._fagarae_FL-isolate
Wolbachia_L._sigmodontis
Wolbachia_L._striatellus
Wolbachia_N._lugens
Wolbachia_S._picta
Wolbachia_W._pumilae
Consensus

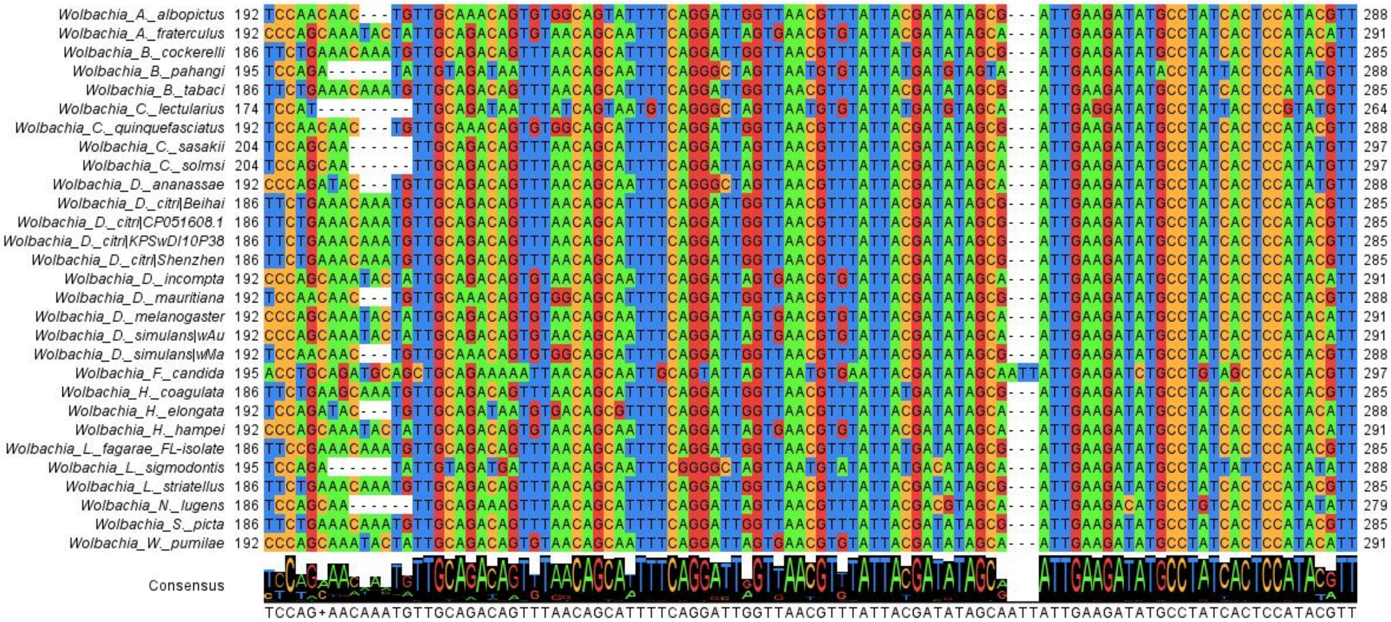
Wolbachia_A._albopictus
Wolbachia_A._fraterculus
Wolbachia_B._cockerelli
Wolbachia_B._pahangi
Wolbachia_B._tabaci
Wolbachia_C._lectularius
Wolbachia_C._quinquefasciatus
Wolbachia_C._sasakii
Wolbachia_C._solmsi
Wolbachia_D._ananassae
Wolbachia_D._citri|Beihai
Wolbachia_D._citri|CP051608.1
Wolbachia_D._citri|KPSwDI10P38
Wolbachia_D._citri|Shenzhen
Wolbachia_D._incompta
Wolbachia_D._mauritiana
Wolbachia_D._melanogaster
Wolbachia_D._simulans|wAu
Wolbachia_D._simulans|wMa
Wolbachia_F._candida
Wolbachia_H._coagulata
Wolbachia_H._elongata
Wolbachia_H._hampei
Wolbachia_L._fagarae_FL-isolate
Wolbachia_L._sigmodontis
Wolbachia_L._striatellus
Wolbachia_N._lugens
Wolbachia_S._picta
Wolbachia_W._pumilae
Consensus

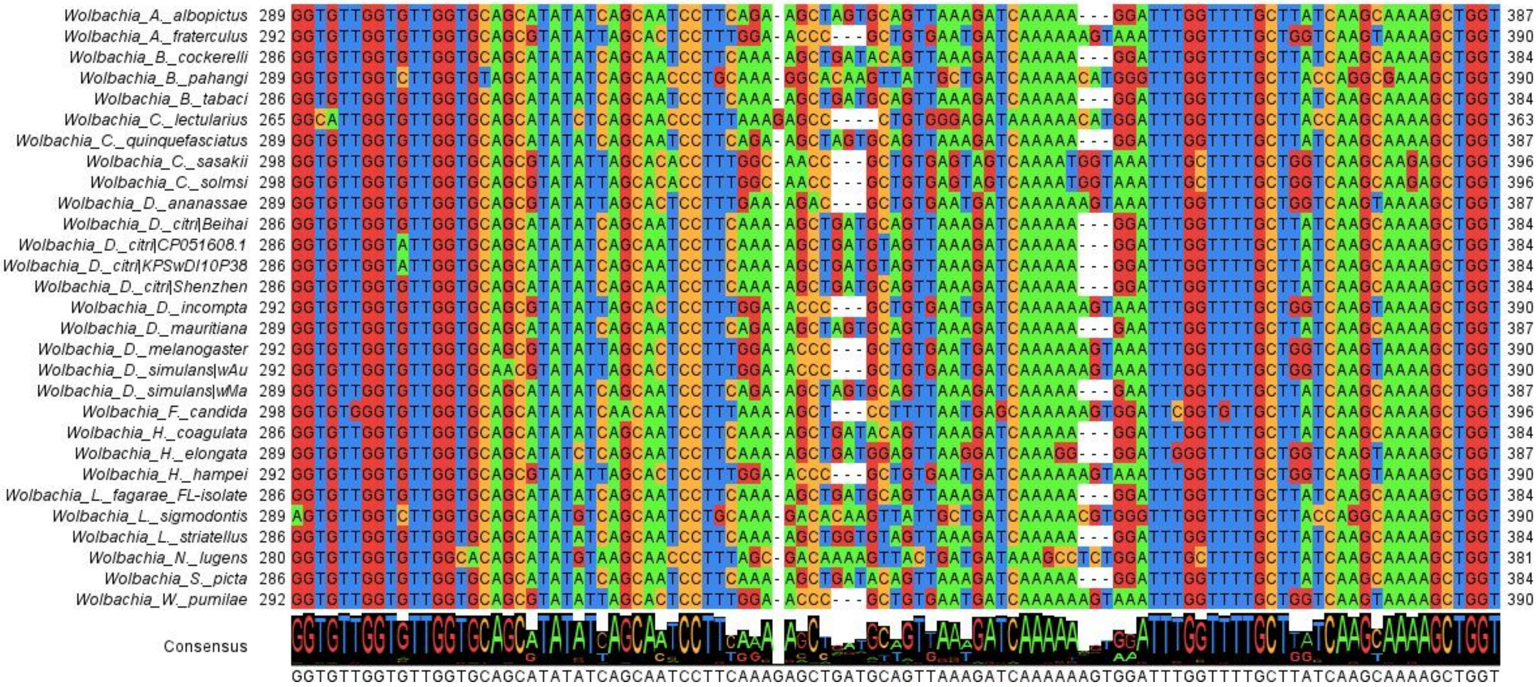
Wolbachia_A._albopictus 289 387
Wolbachia_A._fraterculus 292 390
Wolbachia_B._cockerelli 286 384
Wolbachia_B._pahangi 289 390
Wolbachia_B._tabaci 286 384
Wolbachia_C._lectularius 265 363
Wolbachia_C._quinquefasciatus 289 387
Wolbachia_C._sasakii 298 396
Wolbachia_C._solmsi 298 396
Wolbachia_D._ananassae 289 387
Wolbachia_D._citri|Beihai 286 384
Wolbachia_D._citri|CP051608.1 286 384
Wolbachia_D._citri|KPSwDI10P38 286 384
Wolbachia_D._citri|Shenzhen 286 384
Wolbachia_D._incompta 292 390
Wolbachia_D._mauritiana 289 387
Wolbachia_D._melanogaster 292 390
Wolbachia_D._simulans|wAu 292 390
Wolbachia_D._simulans|wMa 289 387
Wolbachia_F._candida 298 396
Wolbachia_H._coagulata 286 384
Wolbachia_H._elongata 289 387
Wolbachia_H._hampei 292 390
Wolbachia_L._fagarae_FL-isolate 286 384
Wolbachia_L._sigmodontis 289 390
Wolbachia_L._striatellus 286 384
Wolbachia_N._lugens 280 381
Wolbachia_S._picta 286 384
Wolbachia_W._pumilae 292 390
Consensus
GGTGTTGGTGTTGGTGCAGCATATATCAGCAATCCTTCAAAGAGCTGATGCAGTTAAAGATCAAAAAAGTGGATTTGGTTTTGCTTATCAAGCAAAAGCTGGT

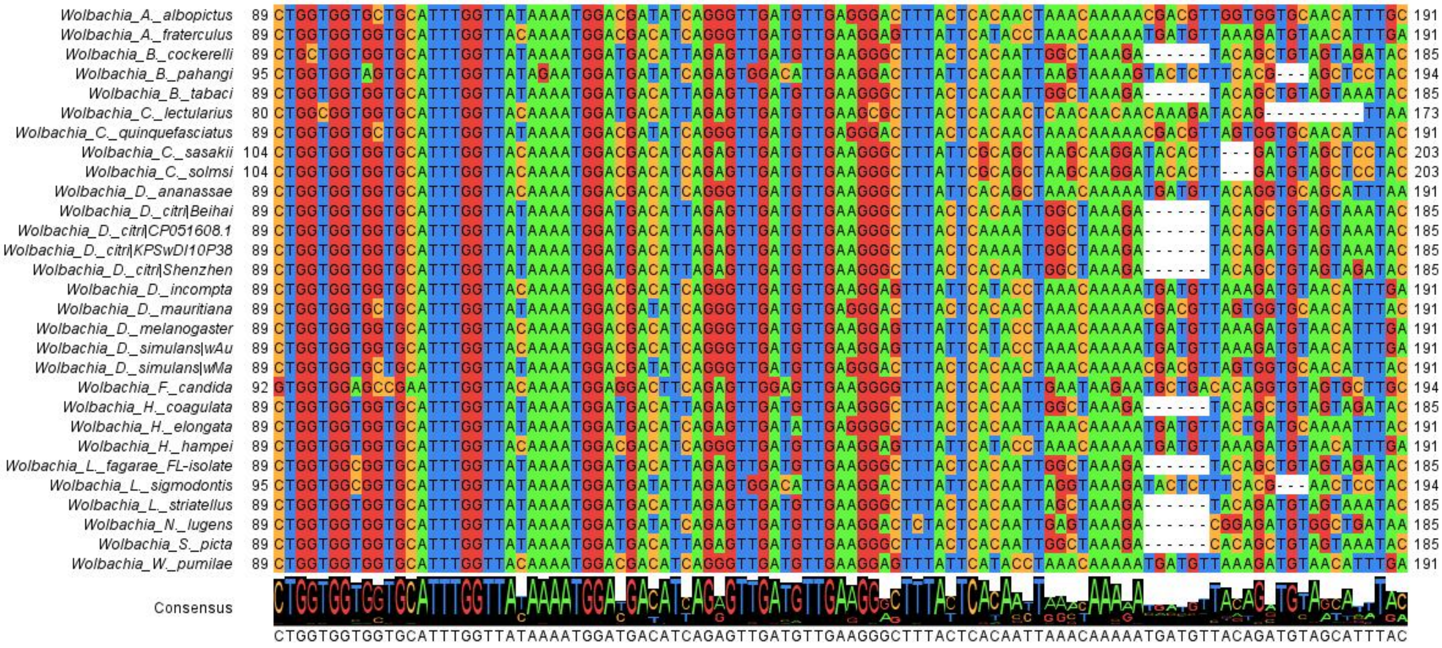
Wolbachia_A._albopictus 89 191
Wolbachia_A._fraterculus 89 191
Wolbachia_B._cockerelli 89 185
Wolbachia_B._pahangi 95 194
Wolbachia_B._tabaci 89 185
Wolbachia_C._lectularius 80 173
Wolbachia_C._quinquefasciatus 89 191
Wolbachia_C._sasakii 104 203
Wolbachia_C._solmsi 104 203
Wolbachia_D._ananassae 89 191
Wolbachia_D._citri|Beihai 89 185
Wolbachia_D._citri|CP051608.1 89 185
Wolbachia_D._citri|KPSwDI10P38 89 185
Wolbachia_D._citri|Shenzhen 89 185
Wolbachia_D._incompta 89 191
Wolbachia_D._mauritiana 89 191
Wolbachia_D._melanogaster 89 191
Wolbachia_D._simulans|wAu 89 191
Wolbachia_D._simulans|wMa 89 191
Wolbachia_F._candida 92 194
Wolbachia_H._coagulata 89 185
Wolbachia_H._elongata 89 191
Wolbachia_H._hampei 89 191
Wolbachia_L._fagarae_FL-isolate 89 185
Wolbachia_L._sigmodontis 95 194
Wolbachia_L._striatellus 89 185
Wolbachia_N._lugens 89 185
Wolbachia_S._picta 89 185
Wolbachia_W._pumilae 89 191
Consensus
CTGGTGGTGGTGCATTTGGTTATAAAATGGATGACATCAGAGTTGATGTTGAAGGGCTTTACTCACAATTAAACAAAAATGATGTTACAGATGTAGCATTTAC

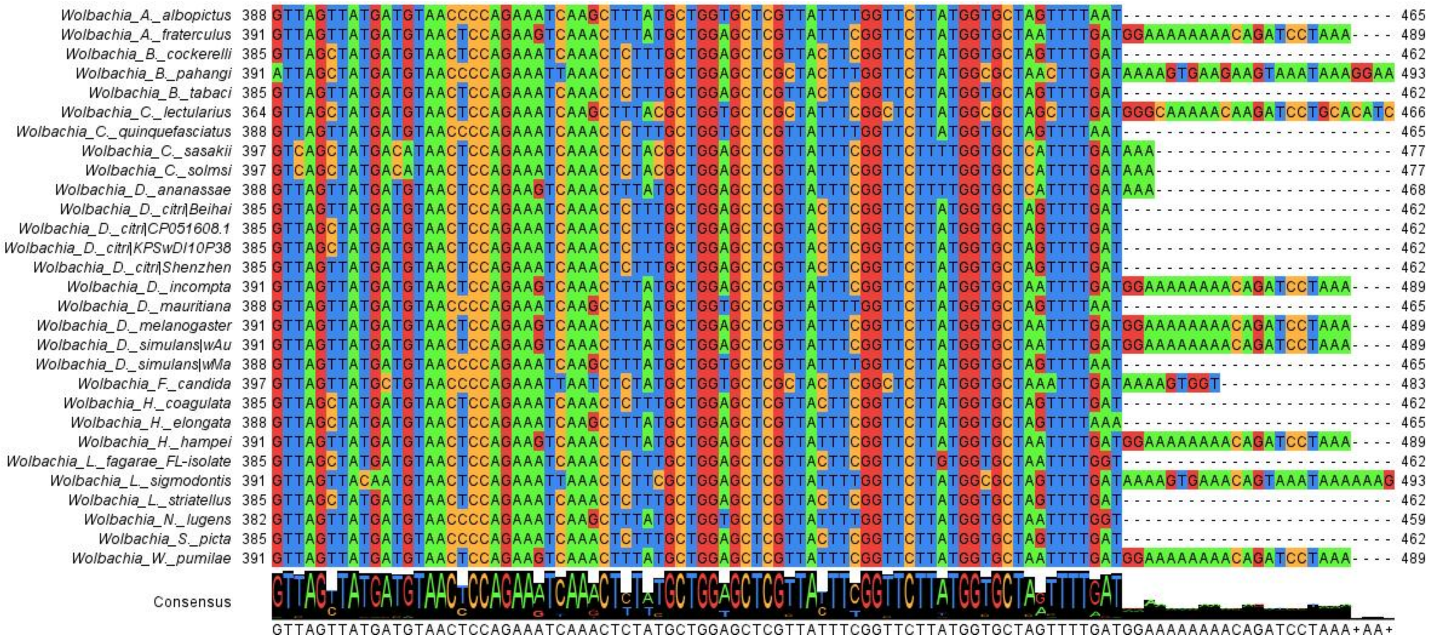

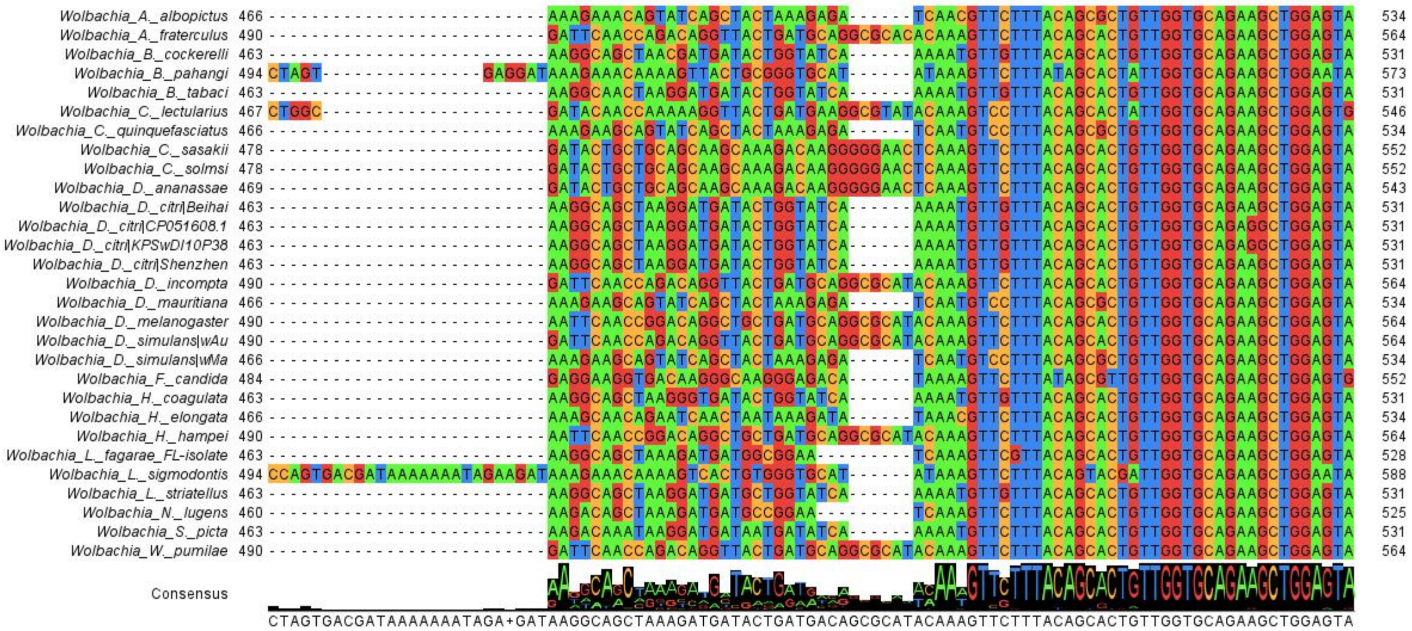

Supplementary Figure 1. Alignment of wsp sequences used to build wsp unrooted maximum likelihood phylogram in Figure 2 of the main text. Sequence alignment performed with MUSCLE in UGENE and visualized in Jalview.

Supplementary Table 1. A complete list of K numbers, assigned by KEGG functional annotation analysis, for all unique wLfag-FL. Transcripts are listed by assigned sequence names and are categorized by 146 pathways

**ko01100 Metabolic pathways (107)**	ko:K00789 metK; S-adenosylmethionine synthetase [EC:2.5.1.6]
ko:K00012 UGDH; UDPglucose 6-dehydrogenase [EC:1.1.1.22]	ko:K00793 ribE; riboflavin synthase [EC:2.5.1.9]
ko:K00024 mdh; malate dehydrogenase [EC:1.1.1.37]	ko:K00812 aspB; aspartate aminotransferase [EC:2.6.1.1]
ko:K00029 E1.1.1.40; malate dehydrogenase (oxaloacetate-decarboxylating)(NADP+) [EC:1.1.1.40]	ko:K00820 glmS; glutamine---fructose-6-phosphate transaminase (isomerizing) [EC:2.6.1.16]
ko:K00031 IDH1; isocitrate dehydrogenase [EC:1.1.1.42]	ko:K00858 ppnK; NAD+ kinase [EC:2.7.1.23]
ko:K00059 fabG; 3-oxoacyl-[acyl-carrier protein] reductase [EC:1.1.1.100]	ko:K00919 ispE; 4-diphosphocytidyl-2-C-methyl-D-erythritol kinase [EC:2.7.1.148]
ko:K00088 IMPDH; IMP dehydrogenase [EC:1.1.1.205]	ko:K00930 argB; acetylglutamate kinase [EC:2.7.2.8]
ko:K00099 dxr; 1-deoxy-D-xylulose-5-phosphate reductoisomerase [EC:1.1.1.267]	ko:K00939 adk; adenylate kinase [EC:2.7.4.3]
ko:K00133 asd; aspartate-semialdehyde dehydrogenase [EC:1.2.1.11]	ko:K00940 ndk; nucleoside-diphosphate kinase [EC:2.7.4.6]
ko:K00161 PDHA; pyruvate dehydrogenase E1 component alpha subunit [EC:1.2.4.1]	ko:K00943 tmk; dTMP kinase [EC:2.7.4.9]
ko:K00162 PDHB; pyruvate dehydrogenase E1 component beta subunit [EC:1.2.4.1]	ko:K00948 PRPS; ribose-phosphate pyrophosphokinase [EC:2.7.6.1]
ko:K00164 OGDH; 2-oxoglutarate dehydrogenase E1 component [EC:1.2.4.2]	ko:K00954 E2.7.7.3A; pantetheine-phosphate adenylyltransferase [EC:2.7.7.3]
ko:K00215 dapB; 4-hydroxy-tetrahydrodipicolinate reductase [EC:1.17.1.8]	ko:K01179 E3.2.1.4; endoglucanase [EC:3.2.1.4]
ko:K00228 CPOX; coproporphyrinogen III oxidase [EC:1.3.3.3]	ko:K01255 CARP; leucyl aminopeptidase [EC:3.4.11.1]
ko:K00239 sdhA; succinate dehydrogenase / fumarate reductase, flavoprotein subunit [EC:1.3.5.1 1.3.5.4]	ko:K01465 URA4; dihydroorotase [EC:3.5.2.3]
ko:K00241 sdhC; succinate dehydrogenase / fumarate reductase, cytochrome b subunit	ko:K01494 dcd; dCTP deaminase [EC:3.5.4.13]
ko:K00330 nuoA; NADH-quinone oxidoreductase subunit A [EC:7.1.1.2]	ko:K01497 ribA; GTP cyclohydrolase II [EC:3.5.4.25]
ko:K00333 nuoD; NADH-quinone oxidoreductase subunit D [EC:7.1.1.2]	ko:K01578 MLYCD; malonyl-CoA decarboxylase [EC:4.1.1.9]
ko:K00334 nuoE; NADH-quinone oxidoreductase subunit E [EC:7.1.1.2]	ko:K01588 purE; 5-(carboxyamino)imidazole ribonucleotide mutase [EC:5.4.99.18]
ko:K00336 nuoG; NADH-quinone oxidoreductase subunit G [EC:7.1.1.2]	ko:K01589 purK; 5-(carboxyamino)imidazole ribonucleotide synthase [EC:6.3.4.18]
ko:K00338 nuoI; NADH-quinone oxidoreductase subunit I [EC:7.1.1.2]	ko:K01647 CS; citrate synthase [EC:2.3.3.1]
ko:K00339 nuoJ; NADH-quinone oxidoreductase subunit J [EC:7.1.1.2]	ko:K01679 E4.2.1.2B; fumarate hydratase, class II [EC:4.2.1.2]
ko:K00341 nuoL; NADH-quinone oxidoreductase subunit L [EC:7.1.1.2]	ko:K01714 dapA; 4-hydroxy-tetrahydrodipicolinate synthase [EC:4.3.3.7]
ko:K00343 nuoN; NADH-quinone oxidoreductase subunit N [EC:7.1.1.2]	ko:K01719 hemD; uroporphyrinogen-III synthase [EC:4.2.1.75]
ko:K00382 DLD; dihydrolipoamide dehydrogenase [EC:1.8.1.4]	ko:K01803 TPI; triosephosphate isomerase (TIM) [EC:5.3.1.1]
ko:K00411 UQCRFS1; ubiquinol-cytochrome c reductase iron-sulfur subunit [EC:7.1.1.8]	ko:K01885 EARS; glutamyl-tRNA synthetase [EC:6.1.1.17]
ko:K00412 CYTB; ubiquinol-cytochrome c reductase cytochrome b subunit	ko:K01902 sucD; succinyl-CoA synthetase alpha subunit [EC:6.2.1.5]
ko:K00449 pcaH; protocatechuate 3,4-dioxygenase, beta subunit [EC:1.13.11.3]	ko:K01903 sucC; succinyl-CoA synthetase beta subunit [EC:6.2.1.5]
ko:K00525 E1.17.4.1A; ribonucleoside-diphosphate reductase alpha chain [EC:1.17.4.1]	ko:K01921 ddl; D-alanine-D-alanine ligase [EC:6.3.2.4]
ko:K00526 E1.17.4.1B; ribonucleoside-diphosphate reductase beta chain [EC:1.17.4.1]	ko:K01929 murF; UDP-N-acetylmuramoyl-tripeptide--D-alanyl-D-alanine ligase [EC:6.3.2.10]
ko:K00568 ubiG; 2-polyprenyl-6-hydroxyphenyl methylase / 3-demethylubiquinone-9 3-methyltransferase [EC:2.1.1.222 2.1.1.64]	ko:K01933 purM; phosphoribosylformylglycinamidine cyclo-ligase [EC:6.3.3.1]
ko:K00600 glyA; glycine hydroxymethyltransferase [EC:2.1.2.1]	ko:K01934 MTHFS; 5-formyltetrahydrofolate cyclo-ligase [EC:6.3.3.2]
ko:K00602 purH; phosphoribosylaminoimidazolecarboxamide formyltransferase / IMP cyclohydrolase [EC:2.1.2.3 3.5.4.10]	ko:K01937 pyrG; CTP synthase [EC:6.3.4.2]
ko:K00609 pyrB; aspartate carbamoyltransferase catalytic subunit [EC:2.1.3.2]	ko:K01939 purA; adenylosuccinate synthase [EC:6.3.4.4]
ko:K00627 DLAT; pyruvate dehydrogenase E2 component (dihydrolipoamide acetyltransferase) [EC:2.3.1.12]	ko:K01945 purD; phosphoribosylamine---glycine ligase [EC:6.3.4.13]
ko:K00643 E2.3.1.37; 5-aminolevulinate synthase [EC:2.3.1.37]	ko:K01951 guaA; GMP synthase (glutamine-hydrolysing) [EC:6.3.5.2]
ko:K00645 fabD; [acyl-carrier-protein] S-malonyltransferase [EC:2.3.1.39]	ko:K01955 carB; carbamoyl-phosphate synthase large subunit [EC:6.3.5.5]
ko:K00648 fabH; 3-oxoacyl-[acyl-carrier-protein] synthase III [EC:2.3.1.180]	ko:K01965 PCCA; propionyl-CoA carboxylase alpha chain [EC:6.4.1.3]
ko:K00658 DLST; 2-oxoglutarate dehydrogenase E2 component (dihydrolipoamide succinyltransferase) [EC:2.3.1.61]	ko:K01966 PCCB; propionyl-CoA carboxylase beta chain [EC:6.4.1.3 2.1.3.15]
ko:K02078 acpP; acyl carrier protein	ko:K00164 OGDH; 2-oxoglutarate dehydrogenase E1 component [EC:1.2.4.2]
ko:K02109 ATPF0B; F-type H+-transporting ATPase subunit b	ko:K00215 dapB; 4-hydroxy-tetrahydrodipicolinate reductase [EC:1.17.1.8]

ko:K02110 ATPF0C; F-type H+-transporting ATPase subunit c	ko:K00228 CPOX; coproporphyrinogen III oxidase [EC:1.3.3.3]
ko:K02111 ATPF1A; F-type H+/Na+-transporting ATPase subunit alpha [EC:7.1.2.2 7.2.2.1]	ko:K00239 sdhA; succinate dehydrogenase / fumarate reductase, flavoprotein subunit [EC:1.3.5.1 1.3.5.4]
ko:K02112 ATPF1B; F-type H+/Na+-transporting ATPase subunit beta [EC:7.1.2.2 7.2.2.1]	ko:K00241 sdhC; succinate dehydrogenase / fumarate reductase, cytochrome b subunit
ko:K02113 ATPF1D; F-type H+-transporting ATPase subunit delta	ko:K00382 DLD; dihydrolipoamide dehydrogenase [EC:1.8.1.4]
ko:K02114 ATPF1E; F-type H+-transporting ATPase subunit epsilon	ko:K00568 ubiG; 2-polyprenyl-6-hydroxyphenyl methylase / 3-demethylubiquinone-9 3-methyltransferase [EC:2.1.1.222 2.1.1.64]
ko:K02258 COX11; cytochrome c oxidase assembly protein subunit 11	ko:K00600 glyA; glycine hydroxymethyltransferase [EC:2.1.2.1]
ko:K02274 coxA; cytochrome c oxidase subunit I [EC:7.1.1.9]	ko:K00602 purH; phosphoribosylaminoimidazolecarboxamide formyltransferase / IMP cyclohydrolase [EC:2.1.2.3 3.5.4.10]
ko:K02275 coxB; cytochrome c oxidase subunit II [EC:7.1.1.9]	ko:K00627 DLAT; pyruvate dehydrogenase E2 component (dihydrolipoamide acetyltransferase) [EC:2.3.1.12]
ko:K02276 coxC; cytochrome c oxidase subunit III [EC:7.1.1.9]	ko:K00643 E2.3.1.37; 5-aminolevulinate synthase [EC:2.3.1.37]
ko:K02433 gatA; aspartyl-tRNA(Asn)/glutamyl-tRNA(Gln) amidotransferase subunit A [EC:6.3.5.6 6.3.5.7]	ko:K00645 fabD; [acyl-carrier-protein] S-malonyltransferase [EC:2.3.1.39]
ko:K02434 gatB; aspartyl-tRNA(Asn)/glutamyl-tRNA(Gln) amidotransferase subunit B [EC:6.3.5.6 6.3.5.7]	ko:K00658 DLST; 2-oxoglutarate dehydrogenase E2 component (dihydrolipoamide succinyltransferase) [EC:2.3.1.61]
ko:K02435 gatC; aspartyl-tRNA(Asn)/glutamyl-tRNA(Gln) amidotransferase subunit C [EC:6.3.5.6 6.3.5.7]	ko:K00789 metK; S-adenosylmethionine synthetase [EC:2.5.1.6]
ko:K02858 ribB; 3,4-dihydroxy 2-butanone 4-phosphate synthase [EC:4.1.99.12]	ko:K00793 ribE; riboflavin synthase [EC:2.5.1.9]
ko:K03185 ubiH; 2-octaprenyl-6-methoxyphenol hydroxylase [EC:1.14.13.-]	ko:K00812 aspB; aspartate aminotransferase [EC:2.6.1.1]
ko:K03527 ispH; 4-hydroxy-3-methylbut-2-en-1-yl diphosphate reductase [EC:1.17.7.4]	ko:K00919 ispE; 4-diphosphocytidyl-2-C-methyl-D-erythritol kinase [EC:2.7.1.148]
ko:K03621 plsX; phosphate acyltransferase [EC:2.3.1.274]	ko:K00930 argB; acetylglutamate kinase [EC:2.7.2.8]
ko:K03644 lipA; lipoyl synthase [EC:2.8.1.8]	ko:K00939 adk; adenylate kinase [EC:2.7.4.3]
ko:K03787 surE; 5'-nucleotidase [EC:3.1.3.5]	ko:K00940 ndk; nucleoside-diphosphate kinase [EC:2.7.4.6]
ko:K08973 hemJ; protoporphyrinogen IX oxidase [EC:1.3.99.-]	ko:K00948 PRPS; ribose-phosphate pyrophosphokinase [EC:2.7.6.1]
ko:K09458 fabF; 3-oxoacyl-[acyl-carrier-protein] synthase II [EC:2.3.1.179]	ko:K01497 ribA; GTP cyclohydrolase II [EC:3.5.4.25]
ko:K09903 pyrH; uridylate kinase [EC:2.7.4.22]	ko:K01588 purE; 5-(carboxyamino)imidazole ribonucleotide mutase [EC:5.4.99.18]
ko:K11175 purN; phosphoribosylglycinamide formyltransferase 1 [EC:2.1.2.2]	ko:K01589 purK; 5-(carboxyamino)imidazole ribonucleotide synthase [EC:6.3.4.18]
ko:K11645 fbaB; fructose-bisphosphate aldolase, class I [EC:4.1.2.13]	ko:K01647 CS; citrate synthase [EC:2.3.3.1]
ko:K11753 ribF; riboflavin kinase / FMN adenylyltransferase [EC:2.7.1.26 2.7.7.2]	ko:K01679 E4.2.1.2B; fumarate hydratase, class II [EC:4.2.1.2]
ko:K11754 folC; dihydrofolate synthase / folylpolyglutamate synthase [EC:6.3.2.12 6.3.2.17]	ko:K01714 dapA; 4-hydroxy-tetrahydrodipicolinate synthase [EC:4.3.3.7]
ko:K13821 putA; RHH-type transcriptional regulator, proline utilization regulon repressor / proline dehydrogenase / delta 1-pyrroline-5-carboxylate dehydrogenase [EC:1.5.5.2 1.2.1.88]	ko:K01719 hemD; uroporphyrinogen-III synthase [EC:4.2.1.75]
ko:K15371 GDH2; glutamate dehydrogenase [EC:1.4.1.2]	ko:K01803 TPI; triosephosphate isomerase (TIM) [EC:5.3.1.1]
ko:K17103 CHO1; CDP-diacylglycerol---serine O-phosphatidyltransferase [EC:2.7.8.8]	ko:K01885 EARS; glutamyl-tRNA synthetase [EC:6.1.1.17]
**ko01110 Biosynthesis of secondary metabolites (57)**	ko:K01902 sucD; succinyl-CoA synthetase alpha subunit [EC:6.2.1.5]
ko:K00024 mdh; malate dehydrogenase [EC:1.1.1.37]	ko:K01903 sucC; succinyl-CoA synthetase beta subunit [EC:6.2.1.5]
ko:K00031 IDH1; isocitrate dehydrogenase [EC:1.1.1.42]	ko:K01933 purM; phosphoribosylformylglycinamidine cyclo-ligase [EC:6.3.3.1]
ko:K00059 fabG; 3-oxoacyl-[acyl-carrier protein] reductase [EC:1.1.1.100]	ko:K01945 purD; phosphoribosylamine---glycine ligase [EC:6.3.4.13]
ko:K00088 IMPDH; IMP dehydrogenase [EC:1.1.1.205]	ko:K01965 PCCA; propionyl-CoA carboxylase alpha chain [EC:6.4.1.3]
ko:K00099 dxr; 1-deoxy-D-xylulose-5-phosphate reductoisomerase [EC:1.1.1.267]	ko:K01966 PCCB; propionyl-CoA carboxylase beta chain [EC:6.4.1.3 2.1.3.15]
ko:K00133 asd; aspartate-semialdehyde dehydrogenase [EC:1.2.1.11]	ko:K02078 acpP; acyl carrier protein
ko:K00161 PDHA; pyruvate dehydrogenase E1 component alpha subunit [EC:1.2.4.1]	ko:K02523 ispB; octaprenyl-diphosphate synthase [EC:2.5.1.90]
ko:K00162 PDHB; pyruvate dehydrogenase E1 component beta subunit [EC:1.2.4.1]	ko:K02858 ribB; 3,4-dihydroxy 2-butanone 4-phosphate synthase [EC:4.1.99.12]
ko:K03185 ubiH; 2-octaprenyl-6-methoxyphenol hydroxylase [EC:1.14.13.-]	ko:K11754 folC; dihydrofolate synthase / folylpolyglutamate synthase [EC:6.3.2.12 6.3.2.17]
ko:K03527 ispH; 4-hydroxy-3-methylbut-2-en-1-yl diphosphate reductase [EC:1.17.7.4]	**ko01120 Microbial metabolism in diverse environments (28)**
ko:K03621 plsX; phosphate acyltransferase [EC:2.3.1.274]	ko:K00024 mdh; malate dehydrogenase [EC:1.1.1.37]
ko:K03787 surE; 5'-nucleotidase [EC:3.1.3.5]	ko:K00029 E1.1.1.40; malate dehydrogenase (oxaloacetate-decarboxylating)(NADP+) [EC:1.1.1.40]
ko:K08973 hemJ; protoporphyrinogen IX oxidase [EC:1.3.99.-]	ko:K00031 IDH1; isocitrate dehydrogenase [EC:1.1.1.42]
ko:K11175 purN; phosphoribosylglycinamide formyltransferase 1 [EC:2.1.2.2]	ko:K00133 asd; aspartate-semialdehyde dehydrogenase [EC:1.2.1.11]

ko:K11645 fbaB; fructose-bisphosphate aldolase, class I [EC:4.1.2.13]	ko:K00161 PDHA; pyruvate dehydrogenase E1 component alpha subunit [EC:1.2.4.1]
ko:K11753 ribF; riboflavin kinase / FMN adenylyltransferase [EC:2.7.1.26 2.7.7.2]	ko:K00162 PDHB; pyruvate dehydrogenase E1 component beta subunit [EC:1.2.4.1]
ko:K13821 putA; RHH-type transcriptional regulator, proline utilization regulon repressor / proline dehydrogenase / delta 1-pyrroline-5-carboxylate dehydrogenase [EC:1.5.5.2 1.2.1.88]	ko:K00164 OGDH; 2-oxoglutarate dehydrogenase E1 component [EC:1.2.4.2]
ko:K17103 CHO1; CDP-diacylglycerol---serine O-phosphatidyltransferase [EC:2.7.8.8]	ko:K00215 dapB; 4-hydroxy-tetrahydrodipicolinate reductase [EC:1.17.1.8]
**ko01240 Biosynthesis of cofactors (29)**	ko:K00239 sdhA; succinate dehydrogenase / fumarate reductase, flavoprotein subunit [EC:1.3.5.1 1.3.5.4]
ko:K00012 UGDH; UDPglucose 6-dehydrogenase [EC:1.1.1.22]	ko:K00241 sdhC; succinate dehydrogenase / fumarate reductase, cytochrome b subunit
ko:K00059 fabG; 3-oxoacyl-[acyl-carrier protein] reductase [EC:1.1.1.100]	ko:K00382 DLD; dihydrolipoamide dehydrogenase [EC:1.8.1.4]
ko:K00228 CPOX; coproporphyrinogen III oxidase [EC:1.3.3.3]	ko:K00449 pcaH; protocatechuate 3,4-dioxygenase, beta subunit [EC:1.13.11.3]
ko:K00382 DLD; dihydrolipoamide dehydrogenase [EC:1.8.1.4]	ko:K00600 glyA; glycine hydroxymethyltransferase [EC:2.1.2.1]
ko:K00568 ubiG; 2-polyprenyl-6-hydroxyphenyl methylase / 3-demethylubiquinone-9 3-methyltransferase [EC:2.1.1.222 2.1.1.64]	ko:K00627 DLAT; pyruvate dehydrogenase E2 component (dihydrolipoamide acetyltransferase) [EC:2.3.1.12]
ko:K00600 glyA; glycine hydroxymethyltransferase [EC:2.1.2.1]	ko:K00658 DLST; 2-oxoglutarate dehydrogenase E2 component (dihydrolipoamide succinyltransferase) [EC:2.3.1.61]
ko:K00609 pyrB; aspartate carbamoyltransferase catalytic subunit [EC:2.1.3.2]	ko:K00948 PRPS; ribose-phosphate pyrophosphokinase [EC:2.7.6.1]
ko:K00643 E2.3.1.37; 5-aminolevulinate synthase [EC:2.3.1.37]	ko:K01647 CS; citrate synthase [EC:2.3.3.1]
ko:K00789 metK; S-adenosylmethionine synthetase [EC:2.5.1.6]	ko:K01679 E4.2.1.2B; fumarate hydratase, class II [EC:4.2.1.2]
ko:K00793 ribE; riboflavin synthase [EC:2.5.1.9]	ko:K01714 dapA; 4-hydroxy-tetrahydrodipicolinate synthase [EC:4.3.3.7]
ko:K00858 ppnK; NAD+ kinase [EC:2.7.1.23]	ko:K01719 hemD; uroporphyrinogen-III synthase [EC:4.2.1.75]
ko:K00939 adk; adenylate kinase [EC:2.7.4.3]	ko:K01803 TPI; triosephosphate isomerase (TIM) [EC:5.3.1.1]
ko:K00940 ndk; nucleoside-diphosphate kinase [EC:2.7.4.6]	ko:K01885 EARS; glutamyl-tRNA synthetase [EC:6.1.1.17]
ko:K00954 E2.7.7.3A; pantetheine-phosphate adenylyltransferase [EC:2.7.7.3]	ko:K01902 sucD; succinyl-CoA synthetase alpha subunit [EC:6.2.1.5]
ko:K01465 URA4; dihydroorotase [EC:3.5.2.3]	ko:K01903 sucC; succinyl-CoA synthetase beta subunit [EC:6.2.1.5]
ko:K01497 ribA; GTP cyclohydrolase II [EC:3.5.4.25]	ko:K01965 PCCA; propionyl-CoA carboxylase alpha chain [EC:6.4.1.3]
ko:K01719 hemD; uroporphyrinogen-III synthase [EC:4.2.1.75]	ko:K01966 PCCB; propionyl-CoA carboxylase beta chain [EC:6.4.1.3 2.1.3.15]
ko:K01885 EARS; glutamyl-tRNA synthetase [EC:6.1.1.17]	ko:K11645 fbaB; fructose-bisphosphate aldolase, class I [EC:4.1.2.13]
ko:K01937 pyrG; CTP synthase [EC:6.3.4.2]	ko:K15371 GDH2; glutamate dehydrogenase [EC:1.4.1.2]
ko:K01939 purA; adenylosuccinate synthase [EC:6.3.4.4]	**ko03010 Ribosome (26)**
ko:K01955 carB; carbamoyl-phosphate synthase large subunit [EC:6.3.5.5]	ko:K02863 RP-L1; large subunit ribosomal protein L1
ko:K02858 ribB; 3,4-dihydroxy 2-butanone 4-phosphate synthase [EC:4.1.99.12]	ko:K02871 RP-L13; large subunit ribosomal protein L13
ko:K03185 ubiH; 2-octaprenyl-6-methoxyphenol hydroxylase [EC:1.14.13.-]	ko:K02878 RP-L16; large subunit ribosomal protein L16
ko:K03644 lipA; lipoyl synthase [EC:2.8.1.8]	ko:K02879 RP-L17; large subunit ribosomal protein L17
ko:K08973 hemJ; protoporphyrinogen IX oxidase [EC:1.3.99.-]	ko:K02881 RP-L18; large subunit ribosomal protein L18
ko:K09458 fabF; 3-oxoacyl-[acyl-carrier-protein] synthase II [EC:2.3.1.179]	ko:K02884 RP-L19; large subunit ribosomal protein L19
ko:K09903 pyrH; uridylate kinase [EC:2.7.4.22]	ko:K02886 RP-L2; large subunit ribosomal protein L2
ko:K11753 ribF; riboflavin kinase / FMN adenylyltransferase [EC:2.7.1.26 2.7.7.2]	ko:K02897 RP-L25; large subunit ribosomal protein L25
ko:K02899 RP-L27; large subunit ribosomal protein L27	ko:K02274 coxA; cytochrome c oxidase subunit I [EC:7.1.1.9]
ko:K02902 RP-L28; large subunit ribosomal protein L28	ko:K02275 coxB; cytochrome c oxidase subunit II [EC:7.1.1.9]
ko:K02906 RP-L3; large subunit ribosomal protein L3	ko:K02276 coxC; cytochrome c oxidase subunit III [EC:7.1.1.9]
ko:K02919 RP-L36; large subunit ribosomal protein L36	**ko01200 Carbon metabolism (21)**
ko:K02933 RP-L6; large subunit ribosomal protein L6	ko:K00024 mdh; malate dehydrogenase [EC:1.1.1.37]
ko:K02939 RP-L9; large subunit ribosomal protein L9	ko:K00029 E1.1.1.40; malate dehydrogenase (oxaloacetate-decarboxylating)(NADP+) [EC:1.1.1.40]
ko:K02945 RP-S1; small subunit ribosomal protein S1	ko:K00031 IDH1; isocitrate dehydrogenase [EC:1.1.1.42]
ko:K02950 RP-S12; small subunit ribosomal protein S12	ko:K00161 PDHA; pyruvate dehydrogenase E1 component alpha subunit [EC:1.2.4.1]
ko:K02952 RP-S13; small subunit ribosomal protein S13	ko:K00162 PDHB; pyruvate dehydrogenase E1 component beta subunit [EC:1.2.4.1]
ko:K02956 RP-S15; small subunit ribosomal protein S15	ko:K00164 OGDH; 2-oxoglutarate dehydrogenase E1 component [EC:1.2.4.2]

ko:K02959 RP-S16; small subunit ribosomal protein S16	ko:K00239 sdhA; succinate dehydrogenase / fumarate reductase, flavoprotein subunit [EC:1.3.5.1 1.3.5.4]
ko:K02967 RP-S2; small subunit ribosomal protein S2	ko:K00241 sdhC; succinate dehydrogenase / fumarate reductase, cytochrome b subunit
ko:K02968 RP-S20; small subunit ribosomal protein S20	ko:K00382 DLD; dihydrolipoamide dehydrogenase [EC:1.8.1.4]
ko:K02982 RP-S3; small subunit ribosomal protein S3	ko:K00600 glyA; glycine hydroxymethyltransferase [EC:2.1.2.1]
ko:K02986 RP-S4; small subunit ribosomal protein S4	ko:K00627 DLAT; pyruvate dehydrogenase E2 component (dihydrolipoamide acetyltransferase) [EC:2.3.1.12]
ko:K02988 RP-S5; small subunit ribosomal protein S5	ko:K00658 DLST; 2-oxoglutarate dehydrogenase E2 component (dihydrolipoamide succinyltransferase) [EC:2.3.1.61]
ko:K02990 RP-S6; small subunit ribosomal protein S6	ko:K00948 PRPS; ribose-phosphate pyrophosphokinase [EC:2.7.6.1]
ko:K02994 RP-S8; small subunit ribosomal protein S8	ko:K01647 CS; citrate synthase [EC:2.3.3.1]
**ko00190 Oxidative phosphorylation (23)**	ko:K01679 E4.2.1.2B; fumarate hydratase, class II [EC:4.2.1.2]
ko:K00239 sdhA; succinate dehydrogenase / fumarate reductase, flavoprotein subunit [EC:1.3.5.1 1.3.5.4]	ko:K01803 TPI; triosephosphate isomerase (TIM) [EC:5.3.1.1]
ko:K00241 sdhC; succinate dehydrogenase / fumarate reductase, cytochrome b subunit	ko:K01902 sucD; succinyl-CoA synthetase alpha subunit [EC:6.2.1.5]
ko:K00330 nuoA; NADH-quinone oxidoreductase subunit A [EC:7.1.1.2]	ko:K01903 sucC; succinyl-CoA synthetase beta subunit [EC:6.2.1.5]
ko:K00333 nuoD; NADH-quinone oxidoreductase subunit D [EC:7.1.1.2]	ko:K01965 PCCA; propionyl-CoA carboxylase alpha chain [EC:6.4.1.3]
ko:K00334 nuoE; NADH-quinone oxidoreductase subunit E [EC:7.1.1.2]	ko:K01966 PCCB; propionyl-CoA carboxylase beta chain [EC:6.4.1.3 2.1.3.15]
ko:K00336 nuoG; NADH-quinone oxidoreductase subunit G [EC:7.1.1.2]	ko:K11645 fbaB; fructose-bisphosphate aldolase, class I [EC:4.1.2.13]
ko:K00338 nuoI; NADH-quinone oxidoreductase subunit I [EC:7.1.1.2]	**ko00230 Purine metabolism (15)**
ko:K00339 nuoJ; NADH-quinone oxidoreductase subunit J [EC:7.1.1.2]	ko:K00088 IMPDH; IMP dehydrogenase [EC:1.1.1.205]
ko:K00341 nuoL; NADH-quinone oxidoreductase subunit L [EC:7.1.1.2]	ko:K00525 E1.17.4.1A; ribonucleoside-diphosphate reductase alpha chain [EC:1.17.4.1]
ko:K00343 nuoN; NADH-quinone oxidoreductase subunit N [EC:7.1.1.2]	ko:K00526 E1.17.4.1B; ribonucleoside-diphosphate reductase beta chain [EC:1.17.4.1]
ko:K00411 UQCRFS1; ubiquinol-cytochrome c reductase iron-sulfur subunit [EC:7.1.1.8]	ko:K00602 purH; phosphoribosylaminoimidazolecarboxamide formyltransferase / IMP cyclohydrolase [EC:2.1.2.3 3.5.4.10]
ko:K00412 CYTB; ubiquinol-cytochrome c reductase cytochrome b subunit	ko:K00939 adk; adenylate kinase [EC:2.7.4.3]
ko:K01507 ppa; inorganic pyrophosphatase [EC:3.6.1.1]	ko:K00940 ndk; nucleoside-diphosphate kinase [EC:2.7.4.6]
ko:K02109 ATPF0B; F-type H+-transporting ATPase subunit b	ko:K00948 PRPS; ribose-phosphate pyrophosphokinase [EC:2.7.6.1]
ko:K02110 ATPF0C; F-type H+-transporting ATPase subunit c	ko:K01588 purE; 5-(carboxyamino)imidazole ribonucleotide mutase [EC:5.4.99.18]
ko:K02111 ATPF1A; F-type H+/Na+-transporting ATPase subunit alpha [EC:7.1.2.2 7.2.2.1]	ko:K01589 purK; 5-(carboxyamino)imidazole ribonucleotide synthase [EC:6.3.4.18]
ko:K02112 ATPF1B; F-type H+/Na+-transporting ATPase subunit beta [EC:7.1.2.2 7.2.2.1]	ko:K01933 purM; phosphoribosylformylglycinamidine cyclo-ligase [EC:6.3.3.1]
ko:K02113 ATPF1D; F-type H+-transporting ATPase subunit delta	ko:K01939 purA; adenylosuccinate synthase [EC:6.3.4.4]
ko:K02114 ATPF1E; F-type H+-transporting ATPase subunit epsilon	ko:K01945 purD; phosphoribosylamine---glycine ligase [EC:6.3.4.13]
ko:K02258 COX11; cytochrome c oxidase assembly protein subunit 11	ko:K01951 guaA; GMP synthase (glutamine-hydrolysing) [EC:6.3.5.2]
ko:K03787 surE; 5'-nucleotidase [EC:3.1.3.5]	ko:K01955 carB; carbamoyl-phosphate synthase large subunit [EC:6.3.5.5]
ko:K11175 purN; phosphoribosylglycinamide formyltransferase 1 [EC:2.1.2.2]	ko:K03787 surE; 5'-nucleotidase [EC:3.1.3.5]
**ko00020 Citrate cycle (TCA cycle) (14)**	ko:K09903 pyrH; uridylate kinase [EC:2.7.4.22]
ko:K00024 mdh; malate dehydrogenase [EC:1.1.1.37]	**ko03070 Bacterial secretion system (10)**
ko:K00031 IDH1; isocitrate dehydrogenase [EC:1.1.1.42]	ko:K03070 secA; preprotein translocase subunit SecA [EC:7.4.2.8]
ko:K00161 PDHA; pyruvate dehydrogenase E1 component alpha subunit [EC:1.2.4.1]	ko:K03072 secD; preprotein translocase subunit SecD
ko:K00162 PDHB; pyruvate dehydrogenase E1 component beta subunit [EC:1.2.4.1]	ko:K03073 secE; preprotein translocase subunit SecE
ko:K00164 OGDH; 2-oxoglutarate dehydrogenase E1 component [EC:1.2.4.2]	ko:K03074 secF; preprotein translocase subunit SecF
ko:K00239 sdhA; succinate dehydrogenase / fumarate reductase, flavoprotein subunit [EC:1.3.5.1 1.3.5.4]	ko:K03106 SRP54; signal recognition particle subunit SRP54 [EC:3.6.5.4]
ko:K00241 sdhC; succinate dehydrogenase / fumarate reductase, cytochrome b subunit	ko:K03196 virB11; type IV secretion system protein VirB11 [EC:7.4.2.8]
ko:K00382 DLD; dihydrolipoamide dehydrogenase [EC:1.8.1.4]	ko:K03199 virB4; type IV secretion system protein VirB4 [EC:7.4.2.8]
ko:K00627 DLAT; pyruvate dehydrogenase E2 component (dihydrolipoamide acetyltransferase) [EC:2.3.1.12]	ko:K03201 virB6; type IV secretion system protein VirB6
ko:K00658 DLST; 2-oxoglutarate dehydrogenase E2 component (dihydrolipoamide succinyltransferase) [EC:2.3.1.61]	ko:K03204 virB9; type IV secretion system protein VirB9
ko:K01647 CS; citrate synthase [EC:2.3.3.1]	ko:K12340 tolC; outer membrane protein

ko:K01679 E4.2.1.2B; fumarate hydratase, class II [EC:4.2.1.2]	**ko00970 Aminoacyl-tRNA biosynthesis (10)**
ko:K01902 sucD; succinyl-CoA synthetase alpha subunit [EC:6.2.1.5]	ko:K01867 WARS; tryptophanyl-tRNA synthetase [EC:6.1.1.2]
ko:K01903 sucC; succinyl-CoA synthetase beta subunit [EC:6.2.1.5]	ko:K01870 IARS; isoleucyl-tRNA synthetase [EC:6.1.1.5]
**ko01230 Biosynthesis of amino acids (12)**	ko:K01873 VARS; valyl-tRNA synthetase [EC:6.1.1.9]
ko:K00031 IDH1; isocitrate dehydrogenase [EC:1.1.1.42]	ko:K01876 DARS2; aspartyl-tRNA synthetase [EC:6.1.1.12]
ko:K00133 asd; aspartate-semialdehyde dehydrogenase [EC:1.2.1.11]	ko:K01878 glyQ; glycyl-tRNA synthetase alpha chain [EC:6.1.1.14]
ko:K00215 dapB; 4-hydroxy-tetrahydrodipicolinate reductase [EC:1.17.1.8]	ko:K01881 PARS; prolyl-tRNA synthetase [EC:6.1.1.15]
ko:K00600 glyA; glycine hydroxymethyltransferase [EC:2.1.2.1]	ko:K01885 EARS; glutamyl-tRNA synthetase [EC:6.1.1.17]
ko:K00789 metK; S-adenosylmethionine synthetase [EC:2.5.1.6]	ko:K02433 gatA; aspartyl-tRNA(Asn)/glutamyl-tRNA(Gln) amidotransferase subunit A [EC:6.3.5.6 6.3.5.7]
ko:K00812 aspB; aspartate aminotransferase [EC:2.6.1.1]	ko:K02434 gatB; aspartyl-tRNA(Asn)/glutamyl-tRNA(Gln) amidotransferase subunit B [EC:6.3.5.6 6.3.5.7]
ko:K00930 argB; acetylglutamate kinase [EC:2.7.2.8]	ko:K02435 gatC; aspartyl-tRNA(Asn)/glutamyl-tRNA(Gln) amidotransferase subunit C [EC:6.3.5.6 6.3.5.7]
ko:K00948 PRPS; ribose-phosphate pyrophosphokinase [EC:2.7.6.1]	**ko02010 ABC transporters (8)**
ko:K01647 CS; citrate synthase [EC:2.3.3.1]	ko:K02012 afuA; iron(III) transport system substrate-binding protein
ko:K01714 dapA; 4-hydroxy-tetrahydrodipicolinate synthase [EC:4.3.3.7]	ko:K02036 pstB; phosphate transport system ATP-binding protein [EC:7.3.2.1]
ko:K01803 TPI; triosephosphate isomerase (TIM) [EC:5.3.1.1]	ko:K02037 pstC; phosphate transport system permease protein
ko:K11645 fbaB; fructose-bisphosphate aldolase, class I [EC:4.1.2.13]	ko:K02066 mlaE; phospholipid/cholesterol/gamma-HCH transport system permease protein
**ko00240 Pyrimidine metabolism (11)**	ko:K02193 ccmA; heme exporter protein A [EC:7.6.2.5]
ko:K00525 E1.17.4.1A; ribonucleoside-diphosphate reductase alpha chain [EC:1.17.4.1]	ko:K09810 lolD; lipoprotein-releasing system ATP-binding protein [EC:7.6.2.-]
ko:K00526 E1.17.4.1B; ribonucleoside-diphosphate reductase beta chain [EC:1.17.4.1]	ko:K09817 znuC; zinc transport system ATP-binding protein [EC:7.2.2.20]
ko:K00609 pyrB; aspartate carbamoyltransferase catalytic subunit [EC:2.1.3.2]	ko:K12536 hasD; ATP-binding cassette, subfamily C, type I secretion system permease/ATPase
ko:K00940 ndk; nucleoside-diphosphate kinase [EC:2.7.4.6]	**ko00250 Alanine, aspartate and glutamate metabolism (7)**
ko:K00943 tmk; dTMP kinase [EC:2.7.4.9]	ko:K00609 pyrB; aspartate carbamoyltransferase catalytic subunit [EC:2.1.3.2]
ko:K01465 URA4; dihydroorotase [EC:3.5.2.3]	ko:K00812 aspB; aspartate aminotransferase [EC:2.6.1.1]
ko:K01494 dcd; dCTP deaminase [EC:3.5.4.13]	ko:K00820 glmS; glutamine---fructose-6-phosphate transaminase (isomerizing) [EC:2.6.1.16]
ko:K01937 pyrG; CTP synthase [EC:6.3.4.2]	ko:K01939 purA; adenylosuccinate synthase [EC:6.3.4.4]
ko:K01955 carB; carbamoyl-phosphate synthase large subunit [EC:6.3.5.5]	ko:K00162 PDHB; pyruvate dehydrogenase E1 component beta subunit [EC:1.2.4.1]
ko:K13821 putA; RHH-type transcriptional regulator, proline utilization regulon repressor / proline dehydrogenase / delta 1-pyrroline-5-carboxylate dehydrogenase [EC:1.5.5.2 1.2.1.88]	ko:K00382 DLD; dihydrolipoamide dehydrogenase [EC:1.8.1.4]
ko:K15371 GDH2; glutamate dehydrogenase [EC:1.4.1.2]	ko:K00627 DLAT; pyruvate dehydrogenase E2 component (dihydrolipoamide acetyltransferase) [EC:2.3.1.12]
**ko02020 Two-component system (7)**	ko:K01679 E4.2.1.2B; fumarate hydratase, class II [EC:4.2.1.2]
ko:K00411 UQCRFS1; ubiquinol-cytochrome c reductase iron-sulfur subunit [EC:7.1.1.8]	**ko00720 Carbon fixation pathways in prokaryotes (7)**
ko:K00412 CYTB; ubiquinol-cytochrome c reductase cytochrome b subunit	ko:K00024 mdh; malate dehydrogenase [EC:1.1.1.37]
ko:K01179 E3.2.1.4; endoglucanase [EC:3.2.1.4]	ko:K00031 IDH1; isocitrate dehydrogenase [EC:1.1.1.42]
ko:K02313 dnaA; chromosomal replication initiator protein	ko:K00239 sdhA; succinate dehydrogenase / fumarate reductase, flavoprotein subunit [EC:1.3.5.1 1.3.5.4]
ko:K02488 pleD; two-component system, cell cycle response regulator [EC:2.7.7.65]	ko:K00241 sdhC; succinate dehydrogenase / fumarate reductase, cytochrome b subunit
ko:K04771 degP; serine protease Do [EC:3.4.21.107]	ko:K01679 E4.2.1.2B; fumarate hydratase, class II [EC:4.2.1.2]
ko:K12340 tolC; outer membrane protein	ko:K01902 sucD; succinyl-CoA synthetase alpha subunit [EC:6.2.1.5]
**ko03018 RNA degradation (7)**	ko:K01903 sucC; succinyl-CoA synthetase beta subunit [EC:6.2.1.5]
ko:K00962 pnp; polyribonucleotide nucleotidyltransferase [EC:2.7.7.8]	**ko00860 Porphyrin and chlorophyll metabolism (6)**
ko:K03628 rho; transcription termination factor Rho	ko:K00228 CPOX; coproporphyrinogen III oxidase [EC:1.3.3.3]
ko:K04043 dnaK; molecular chaperone DnaK	ko:K00643 E2.3.1.37; 5-aminolevulinate synthase [EC:2.3.1.37]
ko:K04077 groEL; chaperonin GroEL	ko:K01719 hemD; uroporphyrinogen-III synthase [EC:4.2.1.75]
ko:K05592 deaD; ATP-dependent RNA helicase DeaD [EC:3.6.4.13]	ko:K01885 EARS; glutamyl-tRNA synthetase [EC:6.1.1.17]
ko:K08300 rne; ribonuclease E [EC:3.1.26.12]	ko:K03594 bfr; bacterioferritin [EC:1.16.3.1]

ko:K12574 rnj; ribonuclease J [EC:3.1.-.-]	ko:K08973 hemJ; protoporphyrinogen IX oxidase [EC:1.3.99.-]
**ko04112 Cell cycle - Caulobacter (7)**	**ko03030 DNA replication (6)**
ko:K01338 lon; ATP-dependent Lon protease [EC:3.4.21.53]	ko:K02314 dnaB; replicative DNA helicase [EC:3.6.4.12]
ko:K01358 clpP; ATP-dependent Clp protease, protease subunit [EC:3.4.21.92]	ko:K02338 dnaN; DNA polymerase III subunit beta [EC:2.7.7.7]
ko:K02313 dnaA; chromosomal replication initiator protein	ko:K02340 holA; DNA polymerase III subunit delta [EC:2.7.7.7]
ko:K02314 dnaB; replicative DNA helicase [EC:3.6.4.12]	ko:K02343 dnaX; DNA polymerase III subunit gamma/tau [EC:2.7.7.7]
ko:K02488 pleD; two-component system, cell cycle response regulator [EC:2.7.7.65]	ko:K03111 ssb; single-strand DNA-binding protein
ko:K03531 ftsZ; cell division protein FtsZ	ko:K03469 rnhA; ribonuclease HI [EC:3.1.26.4]
ko:K03544 clpX; ATP-dependent Clp protease ATP-binding subunit ClpX	**ko00195 Photosynthesis (6)**
**ko03430 Mismatch repair (7)**	ko:K02109 ATPF0B; F-type H+-transporting ATPase subunit b
ko:K02338 dnaN; DNA polymerase III subunit beta [EC:2.7.7.7]	ko:K02110 ATPF0C; F-type H+-transporting ATPase subunit c
ko:K02340 holA; DNA polymerase III subunit delta [EC:2.7.7.7]	ko:K02111 ATPF1A; F-type H+/Na+-transporting ATPase subunit alpha [EC:7.1.2.2 7.2.2.1]
ko:K02343 dnaX; DNA polymerase III subunit gamma/tau [EC:2.7.7.7]	ko:K02112 ATPF1B; F-type H+/Na+-transporting ATPase subunit beta [EC:7.1.2.2 7.2.2.1]
ko:K03111 ssb; single-strand DNA-binding protein	ko:K02113 ATPF1D; F-type H+-transporting ATPase subunit delta
ko:K03555 mutS; DNA mismatch repair protein MutS	ko:K02114 ATPF1E; F-type H+-transporting ATPase subunit epsilon
ko:K03572 mutL; DNA mismatch repair protein MutL	**ko00630 Glyoxylate and dicarboxylate metabolism (6)**
ko:K03657 uvrD; DNA helicase II / ATP-dependent DNA helicase PcrA [EC:3.6.4.12]	ko:K00024 mdh; malate dehydrogenase [EC:1.1.1.37]
**ko00620 Pyruvate metabolism (7)**	ko:K00382 DLD; dihydrolipoamide dehydrogenase [EC:1.8.1.4]
ko:K00024 mdh; malate dehydrogenase [EC:1.1.1.37]	ko:K00600 glyA; glycine hydroxymethyltransferase [EC:2.1.2.1]
ko:K00029 E1.1.1.40; malate dehydrogenase (oxaloacetate-decarboxylating)(NADP+) [EC:1.1.1.40]	ko:K01647 CS; citrate synthase [EC:2.3.3.1]
ko:K00161 PDHA; pyruvate dehydrogenase E1 component alpha subunit [EC:1.2.4.1]	ko:K01965 PCCA; propionyl-CoA carboxylase alpha chain [EC:6.4.1.3]
ko:K01966 PCCB; propionyl-CoA carboxylase beta chain [EC:6.4.1.3 2.1.3.15]	**ko03060 Protein export (5)**
**ko00010 Glycolysis / Gluconeogenesis (6)**	ko:K03070 secA; preprotein translocase subunit SecA [EC:7.4.2.8]
ko:K00161 PDHA; pyruvate dehydrogenase E1 component alpha subunit [EC:1.2.4.1]	ko:K03072 secD; preprotein translocase subunit SecD
ko:K00162 PDHB; pyruvate dehydrogenase E1 component beta subunit [EC:1.2.4.1]	ko:K03073 secE; preprotein translocase subunit SecE
ko:K00382 DLD; dihydrolipoamide dehydrogenase [EC:1.8.1.4]	ko:K03074 secF; preprotein translocase subunit SecF
ko:K00627 DLAT; pyruvate dehydrogenase E2 component (dihydrolipoamide acetyltransferase) [EC:2.3.1.12]	ko:K03106 SRP54; signal recognition particle subunit SRP54 [EC:3.6.5.4]
ko:K01803 TPI; triosephosphate isomerase (TIM) [EC:5.3.1.1]	**ko00260 Glycine, serine and threonine metabolism (5)**
ko:K11645 fbaB; fructose-bisphosphate aldolase, class I [EC:4.1.2.13]	ko:K00133 asd; aspartate-semialdehyde dehydrogenase [EC:1.2.1.11]
**ko00640 Propanoate metabolism (6)**	ko:K00382 DLD; dihydrolipoamide dehydrogenase [EC:1.8.1.4]
ko:K00382 DLD; dihydrolipoamide dehydrogenase [EC:1.8.1.4]	ko:K00600 glyA; glycine hydroxymethyltransferase [EC:2.1.2.1]
ko:K01578 MLYCD; malonyl-CoA decarboxylase [EC:4.1.1.9]	ko:K00643 E2.3.1.37; 5-aminolevulinate synthase [EC:2.3.1.37]
ko:K01902 sucD; succinyl-CoA synthetase alpha subunit [EC:6.2.1.5]	ko:K17103 CHO1; CDP-diacylglycerol---serine O-phosphatidyltransferase [EC:2.7.8.8]
ko:K01903 sucC; succinyl-CoA synthetase beta subunit [EC:6.2.1.5]	**ko00300 Lysine biosynthesis (4)**
ko:K01965 PCCA; propionyl-CoA carboxylase alpha chain [EC:6.4.1.3]	ko:K00133 asd; aspartate-semialdehyde dehydrogenase [EC:1.2.1.11]
ko:K01966 PCCB; propionyl-CoA carboxylase beta chain [EC:6.4.1.3 2.1.3.15]	ko:K00215 dapB; 4-hydroxy-tetrahydrodipicolinate reductase [EC:1.17.1.8]
**ko01210 2-Oxocarboxylic acid metabolism (5)**	ko:K01714 dapA; 4-hydroxy-tetrahydrodipicolinate synthase [EC:4.3.3.7]
ko:K00031 IDH1; isocitrate dehydrogenase [EC:1.1.1.42]	ko:K01929 murF; UDP-N-acetylmuramoyl-tripeptide--D-alanyl-D-alanine ligase [EC:6.3.2.10]
ko:K00133 asd; aspartate-semialdehyde dehydrogenase [EC:1.2.1.11]	**ko00061 Fatty acid biosynthesis (4)**
ko:K00812 aspB; aspartate aminotransferase [EC:2.6.1.1]	ko:K00059 fabG; 3-oxoacyl-[acyl-carrier protein] reductase [EC:1.1.1.100]
ko:K00930 argB; acetylglutamate kinase [EC:2.7.2.8]	ko:K00645 fabD; [acyl-carrier-protein] S-malonyltransferase [EC:2.3.1.39]
ko:K01647 CS; citrate synthase [EC:2.3.3.1]	ko:K00648 fabH; 3-oxoacyl-[acyl-carrier-protein] synthase III [EC:2.3.1.180]
**ko03440 Homologous recombination (5)**	ko:K09458 fabF; 3-oxoacyl-[acyl-carrier-protein] synthase II [EC:2.3.1.179]
ko:K02338 dnaN; DNA polymerase III subunit beta [EC:2.7.7.7]	**ko00900 Terpenoid backbone biosynthesis (4)**

ko:K02340 holA; DNA polymerase III subunit delta [EC:2.7.7.7]	ko:K00099 dxr; 1-deoxy-D-xylulose-5-phosphate reductoisomerase [EC:1.1.1.267]
ko:K02343 dnaX; DNA polymerase III subunit gamma/tau [EC:2.7.7.7]	ko:K00919 ispE; 4-diphosphocytidyl-2-C-methyl-D-erythritol kinase [EC:2.7.1.148]
ko:K03111 ssb; single-strand DNA-binding protein	ko:K02523 ispB; octaprenyl-diphosphate synthase [EC:2.5.1.90]
ko:K03553 recA; recombination protein RecA	ko:K03527 ispH; 4-hydroxy-3-methylbut-2-en-1-yl diphosphate reductase [EC:1.17.7.4]
**ko02024 Quorum sensing (5)**	**ko00710 Carbon fixation in photosynthetic organisms (4)**
ko:K01497 ribA; GTP cyclohydrolase II [EC:3.5.4.25]	ko:K00024 mdh; malate dehydrogenase [EC:1.1.1.37]
ko:K03070 secA; preprotein translocase subunit SecA [EC:7.4.2.8]	ko:K00029 E1.1.1.40; malate dehydrogenase (oxaloacetate-decarboxylating)(NADP+) [EC:1.1.1.40]
ko:K03073 secE; preprotein translocase subunit SecE	ko:K01803 TPI; triosephosphate isomerase (TIM) [EC:5.3.1.1]
ko:K03106 SRP54; signal recognition particle subunit SRP54 [EC:3.6.5.4]	ko:K11645 fbaB; fructose-bisphosphate aldolase, class I [EC:4.1.2.13]
ko:K20327 xagB; glycosyltransferase XagB	**ko00270 Cysteine and methionine metabolism (4)**
**ko05415 Diabetic cardiomyopathy (5)**	ko:K00024 mdh; malate dehydrogenase [EC:1.1.1.37]
ko:K00161 PDHA; pyruvate dehydrogenase E1 component alpha subunit [EC:1.2.4.1]	ko:K00133 asd; aspartate-semialdehyde dehydrogenase [EC:1.2.1.11]
ko:K00162 PDHB; pyruvate dehydrogenase E1 component beta subunit [EC:1.2.4.1]	ko:K00789 metK; S-adenosylmethionine synthetase [EC:2.5.1.6]
ko:K00411 UQCRFS1; ubiquinol-cytochrome c reductase iron-sulfur subunit [EC:7.1.1.8]	ko:K00812 aspB; aspartate aminotransferase [EC:2.6.1.1]
ko:K00412 CYTB; ubiquinol-cytochrome c reductase cytochrome b subunit	**ko00670 One carbon pool by folate (4)**
ko:K00820 glmS; glutamine---fructose-6-phosphate transaminase (isomerizing) [EC:2.6.1.16]	ko:K00600 glyA; glycine hydroxymethyltransferase [EC:2.1.2.1]
ko:K00602 purH; phosphoribosylaminoimidazolecarboxamide formyltransferase / IMP cyclohydrolase [EC:2.1.2.3 3.5.4.10]	ko:K00382 DLD; dihydrolipoamide dehydrogenase [EC:1.8.1.4]
ko:K01934 MTHFS; 5-formyltetrahydrofolate cyclo-ligase [EC:6.3.3.2]	ko:K01965 PCCA; propionyl-CoA carboxylase alpha chain [EC:6.4.1.3]
ko:K11175 purN; phosphoribosylglycinamide formyltransferase 1 [EC:2.1.2.2]	ko:K01966 PCCB; propionyl-CoA carboxylase beta chain [EC:6.4.1.3 2.1.3.15]
**ko01212 Fatty acid metabolism (4)**	**ko05208 Chemical carcinogenesis - reactive oxygen species (3)**
ko:K00059 fabG; 3-oxoacyl-[acyl-carrier protein] reductase [EC:1.1.1.100]	ko:K00411 UQCRFS1; ubiquinol-cytochrome c reductase iron-sulfur subunit [EC:7.1.1.8]
ko:K00645 fabD; [acyl-carrier-protein] S-malonyltransferase [EC:2.3.1.39]	ko:K00412 CYTB; ubiquinol-cytochrome c reductase cytochrome b subunit
ko:K00648 fabH; 3-oxoacyl-[acyl-carrier-protein] synthase III [EC:2.3.1.180]	ko:K04564 SOD2; superoxide dismutase, Fe-Mn family [EC:1.15.1.1]
ko:K09458 fabF; 3-oxoacyl-[acyl-carrier-protein] synthase II [EC:2.3.1.179]	**ko00261 Monobactam biosynthesis (3)**
**ko00740 Riboflavin metabolism (4)**	ko:K00133 asd; aspartate-semialdehyde dehydrogenase [EC:1.2.1.11]
ko:K00793 ribE; riboflavin synthase [EC:2.5.1.9]	ko:K00215 dapB; 4-hydroxy-tetrahydrodipicolinate reductase [EC:1.17.1.8]
ko:K01497 ribA; GTP cyclohydrolase II [EC:3.5.4.25]	ko:K01714 dapA; 4-hydroxy-tetrahydrodipicolinate synthase [EC:4.3.3.7]
ko:K02858 ribB; 3,4-dihydroxy 2-butanone 4-phosphate synthase [EC:4.1.99.12]	ko03020 RNA polymerase (3)
ko:K11753 ribF; riboflavin kinase / FMN adenylyltransferase [EC:2.7.1.26 2.7.7.2]	ko:K03040 rpoA; DNA-directed RNA polymerase subunit alpha [EC:2.7.7.6]
**ko04212 Longevity regulating pathway - worm (4)**	ko:K03060 rpoZ; DNA-directed RNA polymerase subunit omega [EC:2.7.7.6]
ko:K01358 clpP; ATP-dependent Clp protease, protease subunit [EC:3.4.21.92]	ko:K13797 rpoBC; DNA-directed RNA polymerase subunit beta-beta' [EC:2.7.7.6]
ko:K04043 dnaK; molecular chaperone DnaK	**ko00983 Drug metabolism - other enzymes (3)**
ko:K04077 groEL; chaperonin GroEL	ko:K00088 IMPDH; IMP dehydrogenase [EC:1.1.1.205]
ko:K04564 SOD2; superoxide dismutase, Fe-Mn family [EC:1.15.1.1]	ko:K00940 ndk; nucleoside-diphosphate kinase [EC:2.7.4.6]
**ko00680 Methane metabolism (3)**	ko:K01951 guaA; GMP synthase (glutamine-hydrolysing) [EC:6.3.5.2]
ko:K00024 mdh; malate dehydrogenase [EC:1.1.1.37]	**ko05230 Central carbon metabolism in cancer (3)**
ko:K00600 glyA; glycine hydroxymethyltransferase [EC:2.1.2.1]	ko:K00031 IDH1; isocitrate dehydrogenase [EC:1.1.1.42]
ko:K11645 fbaB; fructose-bisphosphate aldolase, class I [EC:4.1.2.13]	ko:K00161 PDHA; pyruvate dehydrogenase E1 component alpha subunit [EC:1.2.4.1]
**ko05012 Parkinson disease (3)**	ko:K00162 PDHB; pyruvate dehydrogenase E1 component beta subunit [EC:1.2.4.1]
ko:K00411 UQCRFS1; ubiquinol-cytochrome c reductase iron-sulfur subunit [EC:7.1.1.8]	**ko05016 Huntington disease (3)**
ko:K00412 CYTB; ubiquinol-cytochrome c reductase cytochrome b subunit	ko:K00411 UQCRFS1; ubiquinol-cytochrome c reductase iron-sulfur subunit [EC:7.1.1.8]
ko:K03671 trxA; thioredoxin 1	ko:K00412 CYTB; ubiquinol-cytochrome c reductase cytochrome b subunit
**ko03420 Nucleotide excision repair (3)**	ko:K04564 SOD2; superoxide dismutase, Fe-Mn family [EC:1.15.1.1]
ko:K03657 uvrD; DNA helicase II / ATP-dependent DNA helicase PcrA [EC:3.6.4.12]	**ko04714 Thermogenesis (3)**

ko:K03701 uvrA; excinuclease ABC subunit A	ko:K00411 UQCRFS1; ubiquinol-cytochrome c reductase iron-sulfur subunit [EC:7.1.1.8]
ko:K03702 uvrB; excinuclease ABC subunit B	ko:K00412 CYTB; ubiquinol-cytochrome c reductase cytochrome b subunit
**ko00550 Peptidoglycan biosynthesis (3)**	ko:K02258 COX11; cytochrome c oxidase assembly protein subunit 11
ko:K01921 ddl; D-alanine-D-alanine ligase [EC:6.3.2.4]	**ko04626 Plant-pathogen interaction (3)**
ko:K01929 murF; UDP-N-acetylmuramoyl-tripeptide--D-alanyl-D-alanine ligase [EC:6.3.2.10]	ko:K02358 tuf; elongation factor Tu
ko:K05515 mrdA; penicillin-binding protein 2 [EC:3.4.16.4]	ko:K04079 HSP90A; molecular chaperone HtpG
**ko05417 Lipid and atherosclerosis (3)**	ko:K12340 tolC; outer membrane protein
ko:K04077 groEL; chaperonin GroEL	**ko04146 Peroxisome (3)**
ko:K04079 HSP90A; molecular chaperone HtpG	ko:K00031 IDH1; isocitrate dehydrogenase [EC:1.1.1.42]
ko:K04564 SOD2; superoxide dismutase, Fe-Mn family [EC:1.15.1.1]	ko:K01578 MLYCD; malonyl-CoA decarboxylase [EC:4.1.1.9]
**ko00280 Valine, leucine and isoleucine degradation (3)**	ko:K04564 SOD2; superoxide dismutase, Fe-Mn family [EC:1.15.1.1]
**ko00220 Arginine biosynthesis (3)**	ko:K00412 CYTB; ubiquinol-cytochrome c reductase cytochrome b subunit
ko:K00812 aspB; aspartate aminotransferase [EC:2.6.1.1]	**ko01502 Vancomycin resistance (2)**
ko:K00930 argB; acetylglutamate kinase [EC:2.7.2.8]	ko:K01921 ddl; D-alanine-D-alanine ligase [EC:6.3.2.4]
ko:K15371 GDH2; glutamate dehydrogenase [EC:1.4.1.2]	ko:K01929 murF; UDP-N-acetylmuramoyl-tripeptide--D-alanyl-D-alanine ligase [EC:6.3.2.10]
**ko00760 Nicotinate and nicotinamide metabolism (2)**	**ko00660 C5-Branched dibasic acid metabolism (2)**
ko:K00858 ppnK; NAD+ kinase [EC:2.7.1.23]	ko:K01902 sucD; succinyl-CoA synthetase alpha subunit [EC:6.2.1.5]
ko:K03787 surE; 5'-nucleotidase [EC:3.1.3.5]	ko:K01903 sucC; succinyl-CoA synthetase beta subunit [EC:6.2.1.5]
**ko01523 Antifolate resistance (2)**	**ko04213 Longevity regulating pathway - multiple species (2)**
ko:K00600 glyA; glycine hydroxymethyltransferase [EC:2.1.2.1]	ko:K03695 clpB; ATP-dependent Clp protease ATP-binding subunit ClpB
ko:K00602 purH; phosphoribosylaminoimidazolecarboxamide formyltransferase / IMP cyclohydrolase [EC:2.1.2.3 3.5.4.10]	ko:K04564 SOD2; superoxide dismutase, Fe-Mn family [EC:1.15.1.1]
**ko00480 Glutathione metabolism (2)**	**ko04066 HIF-1 signaling pathway (2)**
ko:K00031 IDH1; isocitrate dehydrogenase [EC:1.1.1.42]	ko:K00161 PDHA; pyruvate dehydrogenase E1 component alpha subunit [EC:1.2.4.1]
ko:K01255 CARP; leucyl aminopeptidase [EC:3.4.11.1]	ko:K00162 PDHB; pyruvate dehydrogenase E1 component beta subunit [EC:1.2.4.1]
**ko05132 Salmonella infection (2)**	**ko00650 Butanoate metabolism (2)**
ko:K03671 trxA; thioredoxin 1	ko:K00239 sdhA; succinate dehydrogenase / fumarate reductase, flavoprotein subunit [EC:1.3.5.1 1.3.5.4]
ko:K04079 HSP90A; molecular chaperone HtpG	ko:K00241 sdhC; succinate dehydrogenase / fumarate reductase, cytochrome b subunit
**ko00051 Fructose and mannose metabolism (2)**	**ko03410 Base excision repair (2)**
ko:K01803 TPI; triosephosphate isomerase (TIM) [EC:5.3.1.1]	ko:K10563 mutM; formamidopyrimidine-DNA glycosylase [EC:3.2.2.23 4.2.99.18]
ko:K11645 fbaB; fructose-bisphosphate aldolase, class I [EC:4.1.2.13]	ko:K10773 NTH; endonuclease III [EC:4.2.99.18]
**ko05134 Legionellosis (2)**	**ko00030 Pentose phosphate pathway (2)**
ko:K00239 sdhA; succinate dehydrogenase / fumarate reductase, flavoprotein subunit [EC:1.3.5.1 1.3.5.4]	ko:K00948 PRPS; ribose-phosphate pyrophosphokinase [EC:2.7.6.1]
ko:K04077 groEL; chaperonin GroEL	ko:K11645 fbaB; fructose-bisphosphate aldolase, class I [EC:4.1.2.13]
**ko00520 Amino sugar and nucleotide sugar metabolism (2)**	**ko00790 Folate biosynthesis (2)**
ko:K00012 UGDH; UDPglucose 6-dehydrogenase [EC:1.1.1.22]	ko:K01497 ribA; GTP cyclohydrolase II [EC:3.5.4.25]
ko:K00820 glmS; glutamine---fructose-6-phosphate transaminase (isomerizing) [EC:2.6.1.16]	ko:K11754 folC; dihydrofolate synthase / folylpolyglutamate synthase [EC:6.3.2.12 6.3.2.17]
**ko04932 Non-alcoholic fatty liver disease (2)**	**ko05200 Pathways in cancer (2)**
ko:K00411 UQCRFS1; ubiquinol-cytochrome c reductase iron-sulfur subunit [EC:7.1.1.8]	ko:K01679 E4.2.1.2B; fumarate hydratase, class II [EC:4.2.1.2]
ko:K00412 CYTB; ubiquinol-cytochrome c reductase cytochrome b subunit	ko:K04079 HSP90A; molecular chaperone HtpG
**ko00333 Prodigiosin biosynthesis (2)**	**ko05020 Prion disease (2)**
ko:K00059 fabG; 3-oxoacyl-[acyl-carrier protein] reductase [EC:1.1.1.100]	ko:K00411 UQCRFS1; ubiquinol-cytochrome c reductase iron-sulfur subunit [EC:7.1.1.8]
ko:K00645 fabD; [acyl-carrier-protein] S-malonyltransferase [EC:2.3.1.39]	ko:K00412 CYTB; ubiquinol-cytochrome c reductase cytochrome b subunit
**ko05010 Alzheimer disease (2)**	**ko00380 Tryptophan metabolism (2)**
ko:K00411 UQCRFS1; ubiquinol-cytochrome c reductase iron-sulfur subunit [EC:7.1.1.8]	ko:K00382 DLD; dihydrolipoamide dehydrogenase [EC:1.8.1.4]

ko:K00412 CYTB; ubiquinol-cytochrome c reductase cytochrome b subunit	ko:K00658 DLST; 2-oxoglutarate dehydrogenase E2 component (dihydrolipoamide succinyltransferase) [EC:2.3.1.61]
**ko01501 beta-Lactam resistance (2)**	**ko05022 Pathways of neurodegeneration - multiple diseases (2)**
ko:K05515 mrdA; penicillin-binding protein 2 [EC:3.4.16.4]	ko:K00411 UQCRFS1; ubiquinol-cytochrome c reductase iron-sulfur subunit [EC:7.1.1.8]
ko:K12340 tolC; outer membrane protein	ko:K00412 CYTB; ubiquinol-cytochrome c reductase cytochrome b subunit
**ko04260 Cardiac muscle contraction (2)**	**ko05152 Tuberculosis (2)**
ko:K00411 UQCRFS1; ubiquinol-cytochrome c reductase iron-sulfur subunit [EC:7.1.1.8]	ko:K04043 dnaK; molecular chaperone DnaK
ko:K04077 groEL; chaperonin GroEL	ko:K15371 GDH2; glutamate dehydrogenase [EC:1.4.1.2]
**ko05418 Fluid shear stress and atherosclerosis (2)**	**ko02040 Flagellar assembly (1)**
ko:K03671 trxA; thioredoxin 1	ko:K03086 rpoD; RNA polymerase primary sigma factor
ko:K04079 HSP90A; molecular chaperone HtpG	**ko00350 Tyrosine metabolism (1)**
**ko00780 Biotin metabolism (2)**	ko:K00812 aspB; aspartate aminotransferase [EC:2.6.1.1]
ko:K00059 fabG; 3-oxoacyl-[acyl-carrier protein] reductase [EC:1.1.1.100]	**ko02026 Biofilm formation - Escherichia coli (1)**
ko:K09458 fabF; 3-oxoacyl-[acyl-carrier-protein] synthase II [EC:2.3.1.179]	ko:K06204 dksA; DnaK suppressor protein
**ko04922 Glucagon signaling pathway (2)**	**ko04122 Sulfur relay system (1)**
ko:K00161 PDHA; pyruvate dehydrogenase E1 component alpha subunit [EC:1.2.4.1]	ko:K00566 mnmA; tRNA-uridine 2-sulfurtransferase [EC:2.8.1.13]
ko:K00162 PDHB; pyruvate dehydrogenase E1 component beta subunit [EC:1.2.4.1]	**ko04152 AMPK signaling pathway (1)**
**ko00310 Lysine degradation (2)**	ko:K01578 MLYCD; malonyl-CoA decarboxylase [EC:4.1.1.9]
ko:K00382 DLD; dihydrolipoamide dehydrogenase [EC:1.8.1.4]	**ko00470 D-Amino acid metabolism (1)**
ko:K00658 DLST; 2-oxoglutarate dehydrogenase E2 component (dihydrolipoamide succinyltransferase) [EC:2.3.1.61]	ko:K01921 ddl; D-alanine-D-alanine ligase [EC:6.3.2.4]
**ko00330 Arginine and proline metabolism (2)**	**ko04931 Insulin resistance (1)**
ko:K00812 aspB; aspartate aminotransferase [EC:2.6.1.1]	ko:K00820 glmS; glutamine---fructose-6-phosphate transaminase (isomerizing) [EC:2.6.1.16]
ko:K13821 putA; RHH-type transcriptional regulator, proline utilization regulon repressor / proline dehydrogenase / delta 1-pyrroline-5-carboxylate dehydrogenase [EC:1.5.5.2 1.2.1.88]	**ko04013 MAPK signaling pathway - fly (1)**
**ko04621 NOD-like receptor signaling pathway (2)**	ko:K04564 SOD2; superoxide dismutase, Fe-Mn family [EC:1.15.1.1]
ko:K03671 trxA; thioredoxin 1	**ko00500 Starch and sucrose metabolism (1)**
ko:K04079 HSP90A; molecular chaperone HtpG	ko:K01179 E3.2.1.4; endoglucanase [EC:3.2.1.4]
**ko05014 Amyotrophic lateral sclerosis (2)**	**ko00730 Thiamine metabolism (1)**
ko:K00411 UQCRFS1; ubiquinol-cytochrome c reductase iron-sulfur subunit [EC:7.1.1.8]	ko:K00939 adk; adenylate kinase [EC:2.7.4.3]
ko:K00412 CYTB; ubiquinol-cytochrome c reductase cytochrome b subunit	**ko04016 MAPK signaling pathway - plant (1)**
**ko00130 Ubiquinone and other terpenoid-quinone biosynthesis (2)**	ko:K00940 ndk; nucleoside-diphosphate kinase [EC:2.7.4.6]
ko:K00568 ubiG; 2-polyprenyl-6-hydroxyphenyl methylase / 3-demethylubiquinone-9 3-methyltransferase [EC:2.1.1.222 2.1.1.64]	**ko05211 Renal cell carcinoma (1)**
ko:K03185 ubiH; 2-octaprenyl-6-methoxyphenol hydroxylase [EC:1.14.13.-]	ko:K01679 E4.2.1.2B; fumarate hydratase, class II [EC:4.2.1.2]
**ko01503 Cationic antimicrobial peptide (CAMP) resistance (2)**	**ko05120 Epithelial cell signaling in Helicobacter pylori infection (1)**
ko:K04771 degP; serine protease Do [EC:3.4.21.107]	ko:K03196 virB11; type IV secretion system protein VirB11 [EC:7.4.2.8]
ko:K12340 tolC; outer membrane protein	**ko00053 Ascorbate and aldarate metabolism (1)**
**ko00562 Inositol phosphate metabolism (1)**	ko:K00012 UGDH; UDPglucose 6-dehydrogenase [EC:1.1.1.22]
ko:K01803 TPI; triosephosphate isomerase (TIM) [EC:5.3.1.1]	**ko00785 Lipoic acid metabolism (1)**
**ko00360 Phenylalanine metabolism (1)**	ko:K03644 lipA; lipoyl synthase [EC:2.8.1.8]
ko:K00812 aspB; aspartate aminotransferase [EC:2.6.1.1]	**ko04211 Longevity regulating pathway (1)**
**ko04914 Progesterone-mediated oocyte maturation (1)**	ko:K04564 SOD2; superoxide dismutase, Fe-Mn family [EC:1.15.1.1]
ko:K04079 HSP90A; molecular chaperone HtpG	**ko00450 Selenocompound metabolism (1)**
**ko05133 Pertussis (1)**	ko:K00384 trxB; thioredoxin reductase (NADPH) [EC:1.8.1.9]
ko:K12340 tolC; outer membrane protein	**ko00410 beta-Alanine metabolism (1)**
**ko00362 Benzoate degradation (1)**	ko:K01578 MLYCD; malonyl-CoA decarboxylase [EC:4.1.1.9]
ko:K00449 pcaH; protocatechuate 3,4-dioxygenase, beta subunit [EC:1.13.11.3]	**ko00040 Pentose and glucuronate interconversions (1)**

**ko00430 Taurine and hypotaurine metabolism (1)**
**ko00561 Glycerolipid metabolism (1)**
ko:K03621 plsX; phosphate acyltransferase [EC:2.3.1.274]
**ko04974 Protein digestion and absorption (1)**
ko:K19721 COL5AS; collagen type V/XI/XXIV/XXVII, alpha
**ko04151 PI3K-Akt signaling pathway (1)**
ko:K04079 HSP90A; molecular chaperone HtpG
**ko04217 Necroptosis (1)**
ko:K04079 HSP90A; molecular chaperone HtpG
**ko05215 Prostate cancer (1)**
ko:K04079 HSP90A; molecular chaperone HtpG
**ko00998 Biosynthesis of various secondary metabolites - part 2 (1)**
ko:K02078 acpP; acyl carrier protein
**ko00460 Cyanoamino acid metabolism (1)**
ko:K00600 glyA; glycine hydroxymethyltransferase [EC:2.1.2.1]
**ko00401 Novobiocin biosynthesis (1)**
ko:K00812 aspB; aspartate aminotransferase [EC:2.6.1.1]
**ko04936 Alcoholic liver disease (1)**
ko:K01578 MLYCD; malonyl-CoA decarboxylase [EC:4.1.1.9]
**ko03320 PPAR signaling pathway (1)**
ko:K00029 E1.1.1.40; malate dehydrogenase (oxaloacetate-decarboxylating)(NADP+) [EC:1.1.1.40]
**ko04141 Protein processing in endoplasmic reticulum (1)**
ko:K04079 HSP90A; molecular chaperone HtpG
**ko05207 Chemical carcinogenesis - receptor activation (1)**
ko:K04079 HSP90A; molecular chaperone HtpG
**ko00564 Glycerophospholipid metabolism (1)**
ko:K17103 CHO1; CDP-diacylglycerol---serine O-phosphatidyltransferase [EC:2.7.8.8]
**ko04934 Cushing syndrome (1)**
ko:K01679 E4.2.1.2B; fumarate hydratase, class II [EC:4.2.1.2]
**ko00541 O-Antigen nucleotide sugar biosynthesis (1)**
ko:K00012 UGDH; UDPglucose 6-dehydrogenase [EC:1.1.1.22]
**ko04940 Type I diabetes mellitus (1)**
ko:K04077 groEL; chaperonin GroEL
**ko04659 Th17 cell differentiation (1)**
ko:K04079 HSP90A; molecular chaperone HtpG
**ko00950 Isoquinoline alkaloid biosynthesis (1)**
ko:K00812 aspB; aspartate aminotransferase [EC:2.6.1.1]
**ko00400 Phenylalanine, tyrosine and tryptophan biosynthesis (1)**
ko:K00812 aspB; aspartate aminotransferase [EC:2.6.1.1]
**ko00910 Nitrogen metabolism (1)**
ko:K00012 UGDH; UDPglucose 6-dehydrogenase [EC:1.1.1.22]
ko:K15371 GDH2; glutamate dehydrogenase [EC:1.4.1.2]
**ko04612 Antigen processing and presentation (1)**
ko:K04079 HSP90A; molecular chaperone HtpG
**ko00770 Pantothenate and CoA biosynthesis (1)**
ko:K00954 E2.7.7.3A; pantetheine-phosphate adenylyltransferase [EC:2.7.7.3]
**ko00624 Polycyclic aromatic hydrocarbon degradation (1)**
ko:K00449 pcaH; protocatechuate 3,4-dioxygenase, beta subunit [EC:1.13.11.3]
**ko04657 IL-17 signaling pathway (1)**
ko:K04079 HSP90A; molecular chaperone HtpG
**ko04068 FoxO signaling pathway (1)**
ko:K04564 SOD2; superoxide dismutase, Fe-Mn family [EC:1.15.1.1]
**ko01220 Degradation of aromatic compounds (1)**
ko:K00449 pcaH; protocatechuate 3,4-dioxygenase, beta subunit [EC:1.13.11.3]
**ko04915 Estrogen signaling pathway (1)**
ko:K04079 HSP90A; molecular chaperone HtpG
**ko00960 Tropane, piperidine and pyridine alkaloid biosynthesis (1)**
ko:K00812 aspB; aspartate aminotransferase [EC:2.6.1.1]

Supplementary Table 2. Accession numbers of sequences used in the alignment bacterioferritin alignment. The three coordination environments (A, B, C) in the ferroxidase center of bacterioferritin are highly conserved, consisting of four alpha helices (A, B, C, and D)

Bacteria Strain	Abbreviation	Accession/PMC ID*
Wolbachia endosymbiont of *Cylisticus convexus*	***w*Ccon**	WP_114517623.1
Wolbachia endosymbiont of *Drosophila melanogaster*	***w*Dmel**	AAS14893.1
Wolbachia endosymbiont of *Diaphorina citri*	***w*Dcit**	QJT96497.1
Cyanobacterium aponinum PCC 10605	***C. apo***	AFZ54996.1
Escherichia coli K-12	***E. coli***	PMC210146*
Azoarcus olearius	***A. ole***	WP_011764351.1
Leptonema illini DSM 21528	***L. ill***	EHQ08100.1
Wolbachia endosymbiont of *Leuronota fagarae*	***w*LfagFL**	-
